# Local Order in the Unfolded State: Conformational Biases and Nearest Neighbor Interactions

**DOI:** 10.3390/biom4030725

**Published:** 2014-07-24

**Authors:** Siobhan Toal, Reinhard Schweitzer-Stenner

**Affiliations:** Department of Chemistry, Drexel University, 3141 Chestnut Street, Philadelphia, PA 19026, USA; E-Mail: rs344@drexel.edu

**Keywords:** unfolded, peptide conformation, pPII, nearest-neighbor, alanine

## Abstract

The discovery of Intrinsically Disordered Proteins, which contain significant levels of disorder yet perform complex biologically functions, as well as unwanted aggregation, has motivated numerous experimental and theoretical studies aimed at describing residue-level conformational ensembles. Multiple lines of evidence gathered over the last 15 years strongly suggest that amino acids residues display unique and restricted conformational preferences in the unfolded state of peptides and proteins, contrary to one of the basic assumptions of the canonical random coil model. To fully understand residue level order/disorder, however, one has to gain a quantitative, experimentally based picture of conformational distributions and to determine the physical basis underlying residue-level conformational biases. Here, we review the experimental, computational and bioinformatic evidence for conformational preferences of amino acid residues in (mostly short) peptides that can be utilized as suitable model systems for unfolded states of peptides and proteins. In this context particular attention is paid to the alleged high polyproline II preference of alanine. We discuss how these conformational propensities may be modulated by peptide solvent interactions and so called nearest-neighbor interactions. The relevance of conformational propensities for the protein folding problem and the understanding of IDPs is briefly discussed.

## 1. Introduction

Over the last few decades, one of the primary goals of protein research has been to more fully understand the driving forces behind protein folding, unfolding, and mis-folding [1,2,3,4,5,6]. In particular, a detailed characterization of the unfolded state of peptides and proteins is necessary for a complete understanding of protein folding/mis-folding processes. The now classical experiments performed by Anfinsen *et al.* showed that proteins can spontaneously and reproducibly fold into their native bio-functional state, indicating that all the information required for protein folding is encoded in the primary amino acid sequence [7]. However, in the well known Levinthal paradox [8], it was countered that proteins fold on time scales far too short (μs-s) to allow for a stochastic search of conformational space, suggesting that the unfolded state is somehow conformationally biased. Anfinsen’s own explanation for this spontaneous re-folding was the so-called “thermodynamic model”, which postulates that the native state is the most thermodynamically stable, and as such the folding process must be a consequence of the drive to minimize the Gibbs free energy. However, considering the vast number of conformational states possible, the question still remained how a protein navigates pathways to this global minimum in energy and how biased the unfolded state is towards the native state [8].

**Figure 1:**
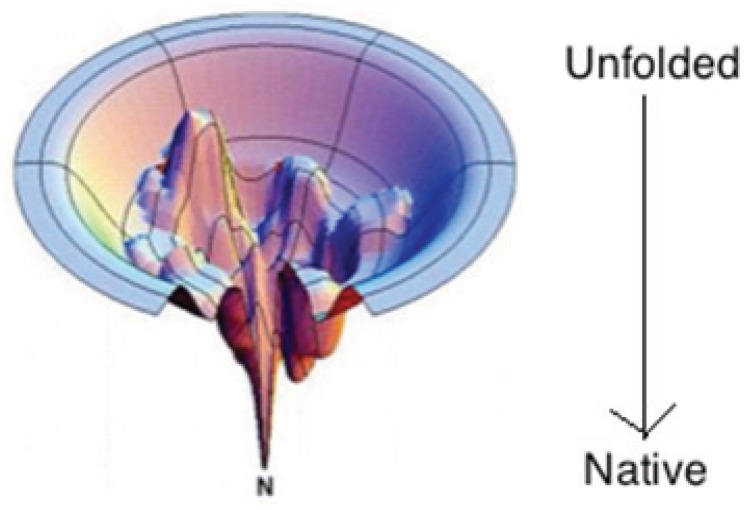
The funneled energy landscape representing the process of going from unfolded state to native state. (Taken from ref. [9] and modified).

Wolynes, Onuchic and colleagues reconsidered Levinthal’s conformational search problem along with Anfinsen’s findings, and took into account that not all conformations are in fact equally likely to be sampled during the folding process [2,9,10]. The so-called “new view” of folding is seen as a flow process of an ensemble of chain molecules. Conformations with lower free energy are more likely then those with higher free energy. The free energy surface of the polypeptide chain can then be viewed as a funnel-like landscape with many local minima corresponding to small energy traps (metastable states) on the order of RT (R: gas constant, T: absolute temperature) and a single global minimum corresponding to the native state (Figure 1). Formation of adventitious contacts and conformations lowers the free energy and increases the likelihood of formation of still further adventitious conformations. This way, the unfolded protein follows a pathway of minimal frustration towards the absolute minimum in Gibbs energy.

Even though the unfolded state constitutes the starting point for this conformational search, it has only recently become the topic of much debate in the scientific community. For a long period of time the unfolded state attracted little scientific attention, in part, due to insufficient methods to study its inherent heterogeneity, but also due to the wide spread notion that it could be described as a so-called “random coil” polymer, the conformational manifold of which is governed solely by steric interactions [11]. Within the aforementioned funnel view, this random coil-like unfolded state can be visualized as a hyperplane of iso-energetic conformations at the top edge of the folding funnel that are governed by large amounts of entropy (see Figure 1). The term “random coil” here deserves further clarification. Within the scope of polymer chemistry a “random coil” generally suggests a long-chain polymer in which the entire backbone exhibits no well-defined structure. This view stems originally from polymer theory, in which a flexible polymer is described as a freely-jointed, freely-rotating chain in a good “theta solvent” in which there are no significant intra-protein, non-local interactions [12,13]. In a theta solvent, the effects of repulsive interactions exactly counterbalance the effects of attractive interactions. Herein we denote this situation as a “global random coil”. The “global random coil” applied to proteins thus assumes that unfolded polypeptide chains adopt a multitude of conformations that lead to a Gaussian distribution of end-to-end distances. This distribution is associated with large conformational and combinatorial entropies, which must be overcome by favorable enthalpic gains (*i.e.*, H-bonding, hydrophobic interactions, *etc.*) in order to fold into a stable state. It is not expected to exhibit any residual structure. The validity of the random coil approach for unfolded and denatured states of proteins was supported by Tanford’s work, where it was shown that denatured proteins have a radius of gyration that conforms to polymeric models of random coils [14]. Specifically, he showed that the intrinsic viscosity, [η], of denatured proteins varies with molecular weight, and hence the number of residues (n), according to a simple power law [η] = n^a^, with the exponent “a” equal to approximately 0.68, well within the range predicted by Flory (0.5–0.8) for random coils [12]. These and similar experiments (e.g., measurements of the radius of gyration by small angle X-ray scattering) probe proteins on a molecular, nanoscale level. Throughout this review, we call the thus probed protein state a “global random coil”. With regard to the sub-nano residue-level scale, Flory’s model for a polymeric random coil assumes that each monomer sub-unit is randomly oriented with respect to neighboring monomer sub-units. This is the so-called “Isolated Pair Hypothesis” (IPH). This hypothesis and Flory’s reliance on an extensive sampling of the Ramachandran space by individual residues [12] are the ingredients of what in the following is called a “local random coil”. This review focuses on an increasing body of experimental, computational and bio-informatic data, which severely challenge the local random coil model while leaving the global random coil model mostly unscathed.

**Figure 2:**
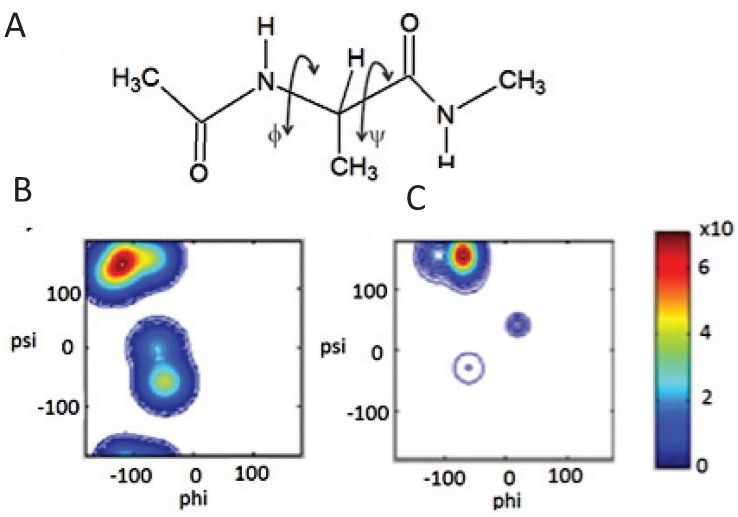
(**A**) The chemical structure of the alanine dipeptide with backbone φ,ψ angles noted; (**B**) The Ramachandran plot depicted the local random coil distribution of the alanine residue of the alanine dipeptide; (**C**) The experimentally obtained Ramachandran plot of the alanine residue of the alanine dipeptide in water as reported by Toal *et al.* [15]*.* (Taken from ref. [15] and modified.

The local random coil situation, in which there is maximal conformational sampling, is illustrated for the alanine dipeptide in Figure 2B [15]. This peptide has been a classical model system for the study of the unfolded state for more than 50 years. For a long period of time since the work of Tanford and Flory the notion that the sterically accessible conformational space is randomly sampled by each residue within an unfolded protein was considered textbook dogma. That greatly suppressed any deeper interest in their exploration. More recently, however, exploring unfolded states has become increasingly relevant due to (1) the observation of residual structure in unfolded states as well as distinguishable conformational biases of individual amino acid residues, which both suggest a breakdown of the local random coil model [16,17,18,19,20,21,22] and (2) the discovery of Intrinsically Disordered Proteins (IDPs), which lack well-defined structure yet perform complex biological functions [1,23,24,25,26]. These proteins have challenged preconceived “lock and key dogma”, which dictated that a protein must fold in order to be bio-functional. Some IDPs are prone to self-aggregation, which causes their involvement in numerous neurodegenerative diseases such as Alzheimer’s, Parkinsons, Huntingtons, the prion diseases, as well as the systemic amyloidosis [27,28,29,30,31,32,33,34,35]. IDPs and unfolded proteins have in common that the structure of both have, until recently, been described as the aforementioned global and/or local random coil polymer. Studying the unfolded state, therefore, should shed light on the key determinants of both the protein folding process and the structural distributions exhibited by various IDPs.

The challenge of the classical random coil model is based on numerous experimental, bioinformatic and to more limited extent computational observations. Firstly, numerous NMR studies have revealed the existence of residual structure in both IDPs and denatured proteins. Works from the laboratories of Dobson and Shortle suggest the existence of some “native-like topology” in the denatured state of proteins, which is a result of non-local interactions [20,21,22,36,37,38,39]. In addition, work by Dyson and Wright [40,41,42,43], and Blackledge, Schwalbe and their associates [16,17,18,19,29,32,44,45,46,47,48] revealed residual structure in the form of local turns as well as unstable helical and strand structures in unfolded peptides and IDPs. Secondly, it is now generally established that individual amino acid residues in short peptides do not conform to the basic assumptions of the local random coil model, in that they display different conformational preferences with a much less entropic Gibbs energy landscape [49,50,51,52]. As will be discussed in detail in Section 2, this notion is particularly true for alanine. Figure 2 illustrates this point by comparing the experimentally based conformational ensemble of the alanine dipeptide (AdP) in water, as recently reported by Toal *et al.* (Figure 2C) [15], with a generated Ramachandran plot depicting a local random coil conformation (Figure 2B). Contrary to classical plots calculated by Ramachandran *et al.* [11] and Flory and coworkers [12] based on steric exclusion models, it is clear that the alanine residue of AdP experimentally shows a rather restricted conformational sampling, which predominantly encompasses the canonical polyproline (pPII) conformational region (Figure 2C). The term “polyproline II” generally refers to the conformation adopted by trans-poly-L-proline which exhibits canonical dihedral angles of (φ,ψ) = (−78°, 145°) located in the upper-left quadrant of the Ramachandran map [53]. As a consequence this polypeptide and closely related proteins, like collagen, thus adopt a left handed 3_1_ helix, which contains three amino acid residues per helical turn, and therefore exhibits three-fold rotational symmetry [54,55,56,57]. The conformational analysis of multiple proteins has revealed that short 3_1_-helical segments exist in many folded proteins, so that pPII deserves to be considered as a secondary structure motif in spite of it being less abundant than α-helices and β-sheets [58,59,60,61,62,63,64,65]. Within the context of this review, however, we do not focus on stable pPII helical structures in folded or globular proteins, and hence the term pPII here reflects solely the transient adoption of the φ and ψ angles in the unfolded state, which correspond to canonical pPII conformations. In Section 2 we detail studies that initially showed the existence of local order in the unfolded state and restricted conformational ensembles of short polypeptides. We move on to discuss the intriguing behavior specifically of alanine with regard to its alleged high pPII preference. While experimental studies have mostly converged to show that alanine has an atypically high pPII preference, some investigations, particularly computational-based studies, have until recently yielded local random coil-like conformations even for alanine [66,67,68,69,70]. However, recent force-field modifications and the use of alternative water models have attempted to move simulated distributions closer to experimental results [71,72].

Although, as we discuss in Section 2, alanine is special with regard to this abnormally high pPII preference in the unfolded state, experimental studies have now converged to reveal that non-alanine residues have dominant though variable pPII/β-strand equilibria, which are much more restricted than local random coil distributions would suggest [52,73,74,75,76,77]. In Section 3, we proceed to discuss data revealing these unique and varied conformational biases of non-alanine residues in detail. Each of the studies mentioned therein at least qualitatively confirms the notion of restricted conformational ensembles for individual amino acid residues in the unfolded state. In addition, a subset of non-proline amino acid residues was recently found to exhibit unexpected high propensities for conformations found in various types of turns [77,78]. Experimentally based evidence for distinguishable conformational preferences is further corroborated by multiple analyses of increasingly large coil libraries, which clearly reveal, for instance, a bias towards pPII-like conformations for alanine residues that are not incorporated in regular secondary structures [75,79,80,81]. In addition to discussing studies on non-alanine peptides in Section 3, we briefly reflect on the current debate on the choice of model systems used for exploring these intrinsic conformational biases, particularly through experimental means. Taken altogether, these results suggest, that conformational ensembles for amino acid residues are varied, unique, and unexplainable in the context of pure steric interaction.

Another point of contention with regard to the random coil model lies in the IPH, *i.e.*, the implicit assumption that conformational sampling of residues are considered independent of the properties and the conformation of their nearest-neighbors in the unfolded state. This idea was first popularized through the work of Flory [12], who showed that each residue’s (φ,ψ) conformation in a polypeptide chain is insensitive to the chemical nature and conformation of neighboring residues. It follows from the IPH that thermodynamic properties of a protein are additive with respect constituent residue along the backbone and that there are large entropic penalties associated with local conformational biases. However, experimental and theoretical evidence that suggests conformational biases has led scientists to also challenge the IPH. Basically, two aspects of the IPH have been questioned. Firstly, theoretical calculations by Pappu *et al.* suggest that the conformational space of nearest neighbors in polyalanine peptides is restricted if a residue for instance adopts right handed helical conformations [82]. Secondly, multiple computational, experimental, and bioinformatic evidence suggest that structural preferences particularly of branched amino acid residues are communicated to the nearest neighbors [81,83,84,85]. If valid, this observation indicates that the conformational ensemble of unfolded proteins/peptides and IDPs cannot be regarded as independent of their amino acid composition. Hence, a physical meaningful description of protein folding and a thorough assessment of the large variety of IDPs require that in addition to intrinsic amino acid conformational propensities, the effect of nearest neighbors is understood in detail. This issue is discussed in detail in Section 4.

In general, this review addresses to what extent results from experimental, bioinformatic and theoretical studies are indicative of the formation of local order in the unfolded state in unfolded peptides and proteins. Local order can be established by strong structural preferences of amino acid residues and by non-local interactions. Herein we focus on the former while keeping the latter in mind. This type of investigation is, however, complicated by experimental limitations and the inherent dynamics of the system, which has in some cases can yield inconsistent results. As previously stated by Kallenbach and associates [49], recent advances have afforded a few systems to become the favored way of studying the unfolded state, namely denatured proteins, intrinsically disordered proteins, charged oligopeptides, short peptides with no long range order, and/or coil libraries. In this review we focus mainly on experimental spectroscopic studies on short peptides and the information they have provided with respect to conformational ensembles and how these are mediated by both nearest-neighbor interactions and solvation. Experimental results obtained for such peptides will be related to conformational propensities derived from various types of coil libraries as well as theoretical methods. In Section 5 we discuss some of the implications that conformational biases in the unfolded state may have for discerning protein structure, folding, as well as for the understanding of IDPs.

## 2. Conformational Propensities in the Unfolded State and the Alanine Debate

### 2.1. Unfolded Does Not Mean Random

The main question concerning unfolded states is whether or not “unfolded” necessarily means “random” or “devoid of secondary structure.” As delineated in the Introduction (Section 1), that is exactly what the random coil model of Tanford, Flory and Ramachandran predicted [11,12,14]. In this view, the unfolded state could be classified locally and globally as a polymeric random-coil, suggesting that each amino acid residue samples all of the sterically allowed regions of the Ramachandran space with nearly equal probability. A visualization of this random sampling of the backbone space is provided in Figure 2 for the alanine dipeptide. Ramachandran *et al.* additionally showed that the superimposed (φ,ψ) distributions of proteins can be approximated by a hard sphere model that considers solely electrostatic repulsion between neighboring atoms [11]. Within this context, the conformational sampling of a residue is considered independent of its nearest neighbor (the IPH) [12].

**Figure 3:**
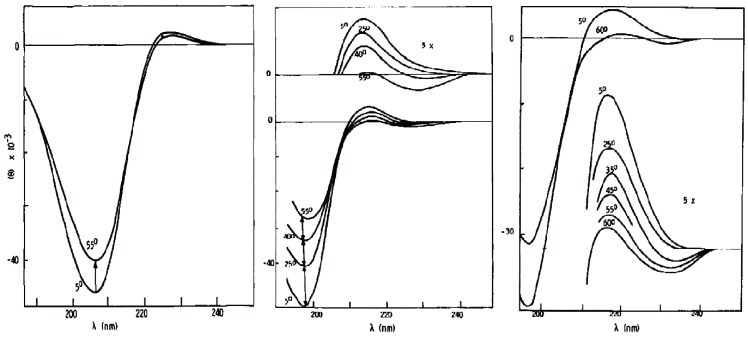
(**left panel**): Ultraviolet circular dichroism (UVCD) spectra of poly-L-proline II, (**middle panel**): poly-L-glutamic acid, and (**right panel**): poly-L-lysine measured as a function of temperature which show a characteristic pPII signals. (Taken and modified form from [87]).

Historically, the local random coil model has not remained unchallenged. Tiffany and Krimm were among the first who questioned its applicability for proteins. In a series of experiments using ultraviolet circular dichroism (UVCD), they showed that the spectrum of unfolded, fully ionized poly-L-glutamic acid and poly-L-lysine are remarkably similar to that of the conformationally restricted trans-L-polyproline (Figure 3) [86,87]. They concluded that charged polypeptides assume, at least locally, a rather ordered pPII conformation. These authors also observed the resemblance between UVCD spectra of proline based peptides (which may be expected to form a stable pPII structure) and proteins unfolded by denaturing agents, which led them to hypothesize that the conformational manifold of unfolded peptides and proteins is dominated by pPII-like conformations. As mentioned within the Introduction (Section 1), in proline rich proteins such as collagen pPII is a rather regular structural motif with backbone dihedral angles (−78°, 146°) [53]. The typical pPII UVCD spectra of non-proline peptides exhibits a pronounced asymmetric couplet with a large negative maximum at approximately 195 nm (π→π*) and small positive maximum at approximately 218 nm (n→π*) (Figure 3). Previous to these studies, this type of UVCD spectrum was strictly associated with disordered chains with no regular secondary structure (*i.e.*, local and global random coils). Woody and coworkers have since shown that the adaptation of pPII conformations in peptides and proteins indeed gives rise to this far UVCD spectrum, although many in the scientific community still misinterpret this signal as indicative of a random coil [88,89,90,91,92].

Tiffany and Krimm’s initial challenge of the random coil model did not at first meet acceptance; [93] at that early time the random coil model and the large number of experiments supporting it were too much engrained in the mind of the protein and peptide communities. The main argument against interpreting the negative couplet in the UVCD spectra of homopeptides as being indicative of pPII was based on the observation that similar couplets were also observed for very short dipeptides in water [93]. Since it was assumed at this time that individual amino acid residues in such short peptides could not exhibit any structural preference, it was concluded that the interpretation of Tiffany and Krimm was questionable.

**Figure 4:**
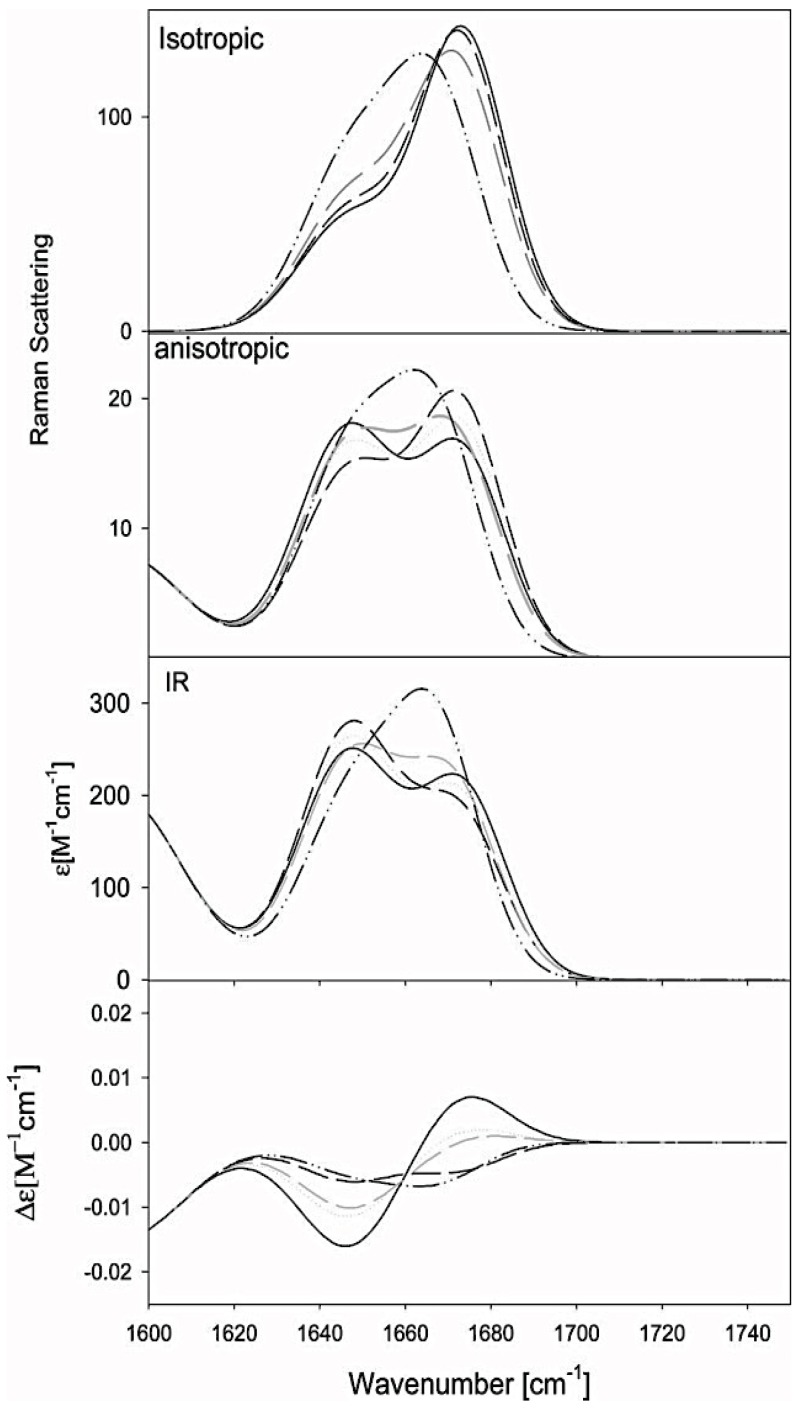
Isotropic Raman, anisotropic Raman, infrared (IR), and vibrational circular dirchroism (VCD) amide I profiles of tripeptides simulated for different conformational ensembles, *i.e.*, 100% pPII (solid line), 100% β-strand (dashed line), 50:50 mixture of pPII and β-strand (dashed gray), and 100% right-handed helical (dashed-dot-dot). (Taken from ref. [101] with permission).

It took nearly 20 years for the questioning of the local random coil model to re-surface. Dukor and Keiderling investigated the same homopeptide as Tiffany and Krimm, but they used vibrational circular dichroism (VCD) rather than electronic UVCD spectroscopy [94]. They showed that the amide I mode of both, poly-prolines (Pro)_n_ of different lengths (*n* = 3, 4) and ionized poly-L-glutamic acid (PLG) give rise to very pronounced negative couplets in the respective VCD spectra, the intensity of which increases with the length of the peptide. Based on this evidence, it was concluded that the conformational ensemble sampled by PLG must in fact have large fractions of pPII, in agreement with Tiffany and Krimm’s work. The VCD of the amide I mode is extremely conformational sensitive and continues to be used as marker by protein biochemists for disentangling various degrees of conformational order [95,96,97,98,99,100,101]. Figure 4 demonstrates this for cationic trialanine [101]. As shown in the bottom panel of Figure 4, the VCD profile for trialanine in 100% pPII conformation displays very pronounced negative-positive couplet centered at the amide I’ frequency, while the intensity of the couplet diminishes upon adding β-strand components, and even reverses sign for purely α-helical conformations. This amide I mode (amide I’ for peptides with deuterated amide groups), which exhibits wavenumbers in the region 1610–1700 cm^−1^, is mainly composed of the CO stretching vibration with admixtures from NH in plane bending (in H_2_O) and CN stretching modes [102,103]. The structural sensitivity of this mode results from coupling between adjacent oscillators, the coupling strength of which depends on the relative orientations of their transition dipole moments and on orientational dependent through bond interactions between local amide I modes in adjacent peptide units [102,104,105,106,107,108]. Thus, the vibrational coupling becomes a function of (φ,ψ) angles of the residues between interacting amide I modes.

These studies, although qualitative in nature, indicated that the unfolded state is not completely “random”, but may exhibit local order due the high preference for pPII-like conformations. What this means in concrete, quantitative terms could not be specified based on the available experimental data. However, this non-mainstream view only entered the mainstream discussion after new studies particularly on alanine based peptides provided more evidence for the notion that the conformational space of amino acid residues is much more restricted than predicted by the local random coil model. The development and the current status of this debate are delineated in the subsequent chapters.

### 2.2. Conformational Preference of Alanine in Water

#### 2.2.1. Experimental Studies

Over the last 20 years short peptides have been extensively utilized as model systems. Generally, such peptides are unable to form the stable hydrogen bonding and long-range interactions needed to fold into well-defined secondary structures. Thus, one can study the conformations of residues in an unfolded state without the necessity of applying denaturing agents, which are likely to directly affect the backbone structure. In particular, short alanine based peptides have been increasingly used in this regard for a variety of reasons. The abundance of alanine in nature, its high propensity for right-handed helices in folded segments of proteins, and its structural simplicity (*i.e.*, a sterically undemanding side chain) render the determination of its conformational propensity in the unfolded state an important step in developing a baseline for exploring structural ensembles formed in the unfolded state of proteins and for understanding the capability of alanine based peptides to form α helices without the scaffolds provided in larger proteins [109,110]. Additionally, short alanine based segments have the practical advantage of being simple enough to allow for direct comparisons between experiment and simulation, thus providing an ultimate testing tool for MD force fields [15,67,72].

**Figure 5:**
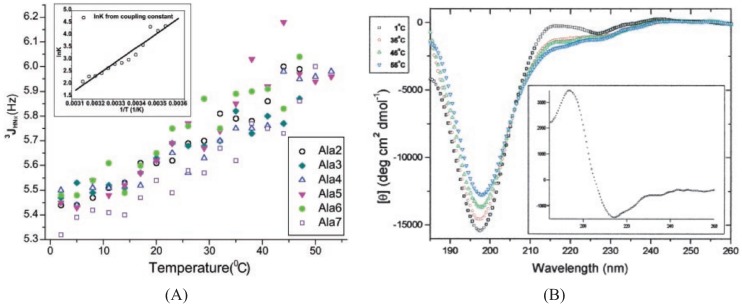
(**A**) The change in ^3^J(H^N^H^α^) with temperature for Ala 2–7 residues of the X_2_A_7_O_2_-NH_2_ peptide; (**B**) The UV-CD spectra of XAO at □ (blue) 1 °C; ○ (green) 35 °C; □ (red) 45 °C; and □ (black) 55 °C. (Taken from ref. [50] and modified).

One of the most notable, and widely debated, studies in this context was performed by Shi *et al.* who utilized HNMR and UVCD spectroscopy to investigate a peptide containing a sequence of seven alanine residues, *i.e.*, X_2_A_7_O_2_-NH_2_ (“XAO”), where O is ornithine and X is diaminoisobutyric acid [50]. Figure 5 shows the structurally sensitive ^3^J(H^N^H^α^) values for all alanine residues within the XAO peptide obtained by these authors as a function of temperature. The ^3^J(H^N^H^α^) coupling constants reflect the spin coupling between the amino and the alpha carbon hydrogens, and hence are sensitive to the intervening φ angle. The monotonous increase of ^3^J(H^N^H^α^) with rising temperatures and the clearly detectable temperature dependence of the CD spectrum indicate that the conformational distribution of the peptide changed as a function of temperature in the region between 0 °C and 52 °C. This would not be the case if most conformations of the ensemble were nearly iso-energetic, as assumed by the ideal local random coil model. The use of the empirical Karplus equation [111,112]:

^3^*J*(*H*^*N*^*H*^*α*^) = *A* cos^2^ (*ϕ*−60^0^ ) + *B* cos(*ϕ*−60^0^ ) + *C* (1)

which relates ^3^J(H^N^H^α^) to the backbone angle ϕ. This φ angle could theoretically correspond to both, pPII and right-handed helical conformations in the upper and lower left quadrants of the Ramachandran plot. However, the measured UVCD spectra (Figure 5) resemble those reported by Tiffany and Krimm [87], thus the authors concluded that pPII dominance is the most likely option. The monotonic increase in ^3^J(H^N^H^α^) coupling constants with rising temperature parallels a decrease of the negative maximum of the measured UVCD spectra and is diagnostic of an increasing population of β-strand-like conformations. The conformation populated at high temperatures must exhibit a positive couplet with a positive signal below and a negative signal above 200 nm, which is diagnostic of β-strand like conformations [113].

The preferential sampling of pPII by alanine in short and unfolded peptides proposed by Kallenbach and colleagues have subsequently been corroborated numerous times by studies on different types of alanine-based peptides [15,114,115,116,117,118,119,120]. In this context, the alanine dipeptide (AdP), which is a single alanine residue flanked by two methyl-blocked peptide groups, has played an important role owing to its traditional use as classical model system since Ramachandran *et al.* used it to construct (φ,ψ) maps 50 years ago [11]. Even prior to the above study on XAO, Han *et al.* reported a strong pPII preference for the AdP peptide based on a comparison of experimental Raman, VCD, and ROA spectra with spectra calculated using DFT approaches [121]. In their study, they mimicked explicit water solvation by constructing AdP complexed to four water molecules (AdP-(H_2_O)_4_) and found that the presence of explicit water imposed a dominant preference for pPII as well as right-handed helical conformations on the alanine residue of the peptide. Weise *et al.* later provided further experimental evidence for pPII dominance in AdP solvated in CsPFO_n_/D_2_O by rationalizing NMR derived dipolar coupling constants with a single representative pPII conformation [122,123]. Using 2D IR experiments, Hochstrasser and co-workers were able to derive the angle between the two amide I’ transition dipoles of AdP which correlates best to a representative pPII-like conformation (−70°, 120°) [124]. More recently, Toal *et al.* used different spectroscopic means to extract realistic (multi-conformational based ensembles) distributions for AdP in water [15]. The result of this study, which is described in more detail below, provides compelling evidence for the predominance of pPII conformation in the conformational ensemble of AdP. In addition to these studies, numerous MD studies on AdP have been carried out, which are independently reviewed below in Subsection 2.2.2 of this chapter (Theoretical Studies on Alanine). Contrary to experimental results, MD studies have yielded mixed results, with regard to the alleged pPII preference of alanine with the majority of the simulations yielded conformational distributions reminiscent of the local random coil picture [15,68,125,126,127,128,129].

In addition to the classic AdP peptide, short unblocked oligoalanines have also been extensively subjected to conformational studies. Woutersen and Hamm, for instance, exploited the backbone sensitivity of the amide I mode in peptides by using non-linear time resolved 2D-IR spectroscopy to analyze cationic trialanine (AAA) in aqueous solution [114,115]. From their experiment they inferred the strength of the nearest neighbor coupling between the peptide’s amide I’ modes and their relative orientation. By combining this with results from *ab initio* calculations on the (φ,ψ)-dependence of excitonic coupling, the authors were able to identify a representative pPII like conformation. In a later study from this group the results of MD simulations were utilized to re-analyze the results of the time resolved IR-experiment; this yielded a 80% fraction for pPII and *ca.* 20% for a right-handed helical conformation [116]. Barron and coworkers took a different approach to investigate the conformational preference of short alanine oligomers [130]. Using Raman optical activity (ROA) measurements, they showed that alanine oligomers from 3–7 residues were predominantly adopting pPII-like structures [102]. To this end, they utilized the appearance of a very specific signal at 1314 cm^−1^, which was found to be diagnostic of pPII. Results from these experiments suggests that alanine oligomers ranging from *tri*- to hepta-alanine prefer pPII conformations in solution and that the pPII content per residue increases with the number of residues. In a separate study, Asher and coworkers used the ψ-sensitivity of one of the UV-resonance enhanced amide III modes to show that a longer helical peptide, referred to as “AP” (AAAAA-(AAARA)_3_-A) unfolds into a predominantly pPII-like structures [131,132].

Work by Schweitzer-Stenner and colleagues have also indicated high pPII preferences for alanine-based peptides. Eker *et al.* for instance, utilized the excitonic coupling between local amide I modes to examine several polyalanines in water [117,118]. For unblocked and semi-blocked tripeptides the authors determined the intensity ratios of the two amide I bands in the respective IR, isotropic, and anisotropic Raman spectra and analyzed them with exciton coupling formalism to determine the (φ,ψ) values of a representative conformation of the peptide’s central residue. Results from this analysis were checked by simulating the corresponding VCD signal for this conformation, which was then compared with the experimentally determined profile. Additional analysis of this representative structure subsequently suggested that trialanine exhibits a 50:50 mixture of pPII and β. A similar investigation was performed on tetraalanine (AAAA) and revealed a higher pPII content [133], in agreement with McColl *et al.* [130].

**Figure 6:**
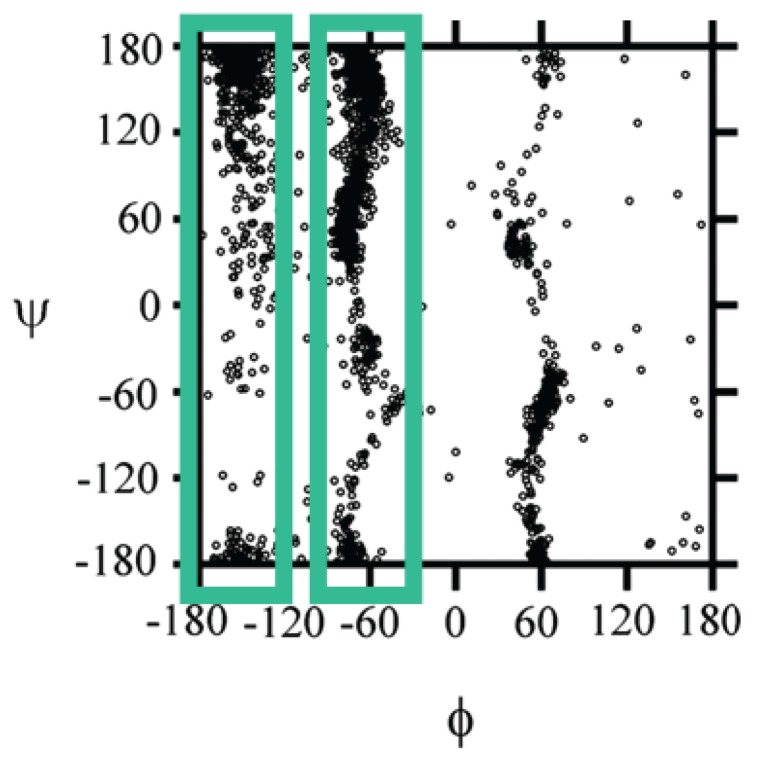
Ramachandran map superimposing the backbone distributions of all residues and all conformational families of the XAO peptide calculated by MD SA with NMR derived time-averaged restraints. The green boxes indicate the two dominant conformational clusters centered at ϕ = −160°, (mainly extended β-strand-like structures) and ϕ = −70° (pPII and β-turn-like conformations). (Taken from ref. [69] and modified).

While the above results qualitatively agree by suggesting a pPII preference of alanine well above the level expected for local random coil distributions (though with different values for pPII fractions), other experimental and computational studies have challenged this notion. Particular in this regard are the conflicting reports concerning the conformational distribution of the aforementioned XAO peptide. In a set of extensive studies, Scheraga, Liwo, and colleagues performed MD simulations using the Amber 99 force field and simulated annealing (MD SA) to predict the conformational ensemble of the XAO peptide [69,70]. The MD simulations were constrained by time averaged distance and angle restraints derived from the authors’ ROE and ^3^J(H^N^H^α^) NMR measurements, respectively [69]. Using this approach they were able to obtain a conformational ensemble consisting of ten dominant families for XAO that satisfy experimental parameters. Figure 6 displays the Ramachandran map these authors obtained, which superimposes the entire MD obtained conformational families of all XAO-residues. From this plot one infer two major sub-populations within the manifold of conformational families, namely one centered at φ = −160 which contains mainly extended β-strand populations, and a second centered at φ = −70 which contains pPII as well as β-turn-like conformations. These results suggest that the peptide exists in an ensemble of inter-converting structures, among which, pPII is only one of many conformations sampled by its alanine residues. These results are supportive of the concept of a statistical coil, which the Scheraga group had earlier suggested as a modified version of the more simplistic random coil model [134,135].

In an effort to resolve the conflicting views of XAO experimentally, Zagrovic *et al.* conducted small angle X-ray scattering (SAXS) measurements on XAO, from which they derived a radius of gyration of 7.4 Å [66]. This value seems to be clearly inconsistent with the substantial sampling of pPII reported by Shi *et al.* [50], for which one would expect a radius of gyration of 11.6 Å. Subsequently, by using the aforementioned MD SA derived conformational ensemble for XAO, Makowska *et al.* [69] were able to reproduce this 7.4 Å radius of gyration obtained by Zagrovic *et al.* as well as the ^3^J(H^N^H^α^) constants of Shi *et al.* with MD simulations, lending further credence to the notion that pPII is not predominantly sampled by the XAO peptide as whole. Interestingly, the conformational manifold of alanine residues derived from this analysis was still somewhat untypical in that it indicated substantial sampling of multiple turn-like conformations, which produced the rather compact structure of the peptide as reflected by its small radius of gyration. Hence, their model is in fact a departure from the classical local random coil.

In an attempt to tackle the conflicting views concerning the conformation of XAO, Schweitzer-Stenner and Measey [136] subsequently utilized the aforementioned structural sensitivity of the amide I mode in polypeptides by simulating the IR, isotropic, anisotropic Raman, and VCD amide I band profiles as well as the ^3^J(H^N^H^α^) constants obtained by Shi *et al.* for XAO [50]. They extended the two-state (pPII↔β) model of Shi *et al.* by constructing a statistical ensemble in which each residue was allowed to adopt a manifold of different representative conformations (pPII, β, helical and various turns structures), thus considering the coil-like results of Makowska *et al.* [69,70] Experimental spectra were fit using these distributions within an excitonic coupling model. As a result, they found that the best reproduction of all experimental data was achieved by assuming an ensemble of conformations which contain various turn (26%) and β-strand conformations (23%) with a sizeable (50%) contribution from canonical pPII conformations. The inclusion of various turn structures at the XA and XO interface of the peptide is generally in agreement with the MD SA derived ensembles of Makowska *et al.* [69], but at variance with the latter. Schweitzer-Stenner and Measey obtained a high pPII contribution localized particularly on the central alanine residues, which clearly suggests an intrinsic pPII preference for alanine, in line with Shi *et al.* With their statistical model, the authors calculated a value of 19.1 Å for the peptide’s end to end distance, which is consistent with the radius of gyration reported by Zagrovic *et al.* [66]. Generally, the authors confirmed the notion that alanine residues exhibit more pPII sampling than predicted by any random or statistical coil models. However, their analysis also hinted to a substantial nearest-neighbor influence of the hydrophilic terminal residues on the conformational manifold of alanine, which is at variance with the IPH of local random coil theory. This and other influences of nearest neighbors will be discussed in more detail in chapter.

**Figure 7:**
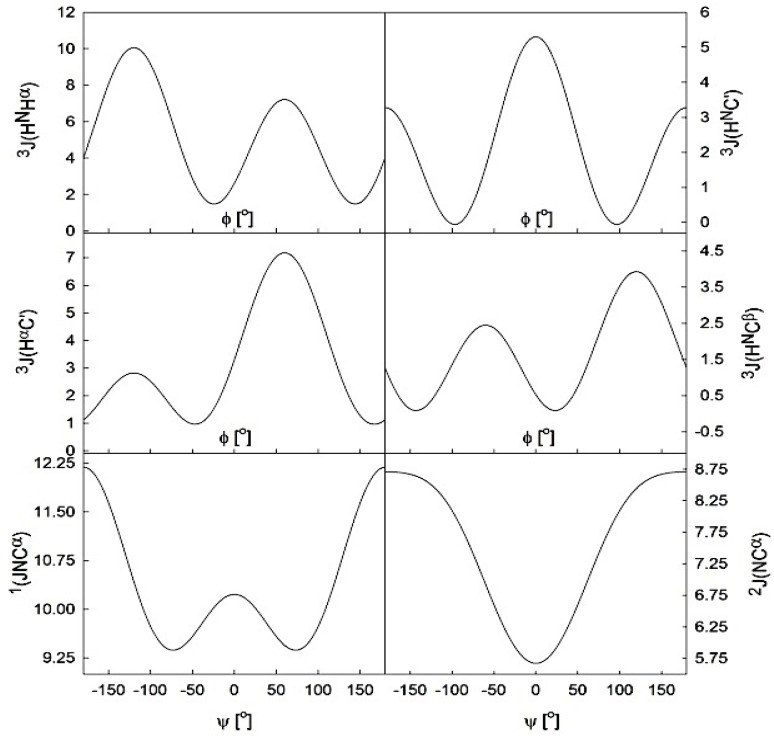
Graphical representation of six Karplus relationships [111,112] of six J-coupling constants which depend differently on the dihedral angles ϕ (**upper and middle panels**) and ψ (**bottom panels**).

Most of the above discussed spectroscopic studies on alanine have in common that they usually invoke “representative” conformations to reproduce experimental data, *i.e.*, single (φ,ψ) pairs for the entire conformational manifold or for subpopulations. Thus they were of limited use for comparisons with conformational distributions obtained from computational studies, for a reliable quantitative assessment of conformational propensities, and for estimating the conformational entropy of unfolded peptides. These shortcomings have been addressed by more recent, increasingly complex studies on the conformational sampling of alanine in short peptides, which combine various techniques like NMR and vibrational spectroscopy as well as theoretical methods to yield a more realistic conformational distributions in the Ramachandran space. The first very important step in this direction was made by Graf *et al.* who combined experimentally derived sets of seven NMR J-coupling constants, each of which relate differently to backbone angles φ and ψ (Figure 7), along with distributions derived from constrained all atom MD simulations [120]. Using this approach, these authors determined that tri- to hepta-alanines predominantly sample pPII (up to 90% for trialanine), with minor admixtures of extended β structure. In agreement with the earlier studies of Shi *et al.* [50], the population of right-handed helical conformations was found to be negligible. As will be discussed below, these results are in sharp conflict with the outcome of MD simulations, and of course, with the local random coil model.

**Figure 8:**
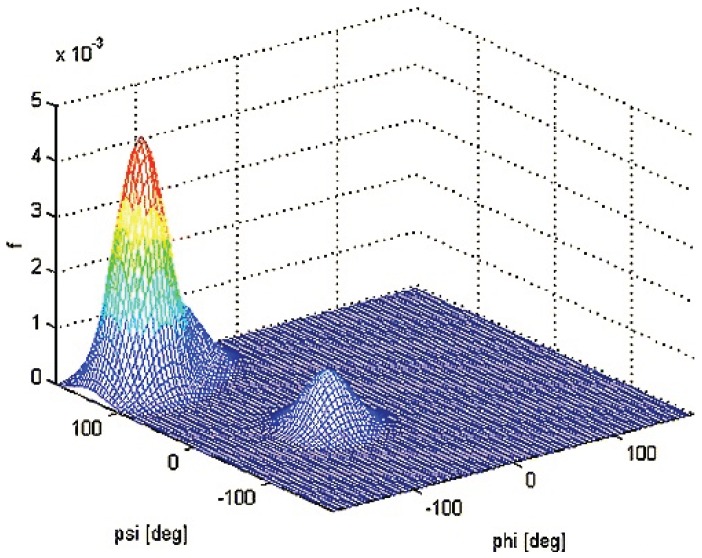
Three-dimensional distribution function in (φ,ψ) space obtained by simulating amide I profiles and NMR coupling constants for trialanine. (Taken from ref. [101] with permission).

Following up on the study of Graf *et al.* [120], Schweitzer-Stenner subsequently used their coupling constants as constraints in a new algorithm which described the conformational ensembles of residues as a superposition of two-dimensional Gaussian functions [101]. This offered a more realistic approach allowing for the width of sub-distributions to be accounted for as compared to the use of representative structures. Using this model, the authors were able to simultaneously reproduce the seven J-coupling constants reported by Graf *et al.* for trialanine as well as the amide I’ band profiles of Raman, IR and VCD reported by Eker *et al.* [118], with a conformational ensemble containing 84% pPII. Thus, they confirmed the high sampling of pPII for this peptide as obtained by Graf *et al.* [120]*.* The conformational distribution function of the central alanine residue of AAA obtained by this study is shown in Figure 8. In this study, the combined use of the structurally very sensitive VCD signal of amide I’ and a large number of φ-dependent coupling constants allowed a rather precise differentiation between pPII and β-strand sub-distributions, whereas the ψ-dependent coupling constants were useful of assessing the relative population of states associated with the upper and lower left quadrant of the Ramachandran plot [101].

While the above referenced studies on short oligoalanines are useful in ascertaining conformational preferences, they also reflect the influence of neighboring alanine residues, and hence, one critical issue that has not been explicitly dealt with, namely, the intrinsic conformational propensity alanine in the absence of any nearest neighbor interactions. Kallenbach and associates addressed this issue by exploring the pPII population of alanine in glycine-based host-guest system AcGGAGGNH2 [137]. The use of glycine-based host-systems is based on the assumption that they provide a minimal steric environment for which to probe the host amino acid’s intrinsic conformational preference. Utilizing an approach reminiscent of previous XAO studies, the authors combined NOE data and ^3^J-coupling constants derived from NMR as well as UVCD to show that alanine in the AcGGAGGNH_2_ peptide predominantly samples pPII, but to a lesser extent than the seven alanine containing XAO peptide [137]. In a subsequent study, Hagarman *et al.* measured and analyzed the J-coupling constants earlier utilized by Graf *et al.* for polyalanines and amide I’ profiles of the unblocked tripeptide GAG [52]. They found a pPII fraction or 79% for alanine, which is slightly lower than the value obtained for AAA (84%). A subsequent paper from this group reported a slightly modified distribution for GAG with a pPII fraction of 0.72 [138]. These studies thus indicate that alanine neighbor indeed stabilizes the pPII conformations slightly. These analyses not only confirmed the high propensity for pPII of a single alanine residue, but also revealed the necessity for more comprehensive intrinsic propensity studies on (non-alanine) residues, which will be discussed in Section 3.

In the context of this chapter, another, somewhat more technical issue shall be briefly addressed. Over the last 10 years, different types of short peptides have been used to explore the conformational propensities of amino acid residues in the unfolded state. Blocked dipeptides are often considered as an ideal choice, owing to the absence of any terminal charges, which are thought to affect the conformation of residues in corresponding unblocked tripeptide systems [73,76,139]. In many of the experimental conformational studies discussed above, blocked and unblocked glycine- and alanine-based peptides have been used. The utilization of the latter is prevalent in vibrational spectroscopy experiments, because the influence of terminal charges guarantee a resolution of the different amide I bands in the IR and Raman spectra of tri- and tetra-peptides [114,116,118,140]. In contrast, blocked peptides have been preferred in many NMR-based studies [141]. Blocked peptides like the classic alanine dipeptide are also the preferred choice for computational studies [125,126,142,143,144]. The question thus arises whether unblocked peptides are really suitable for exact “measurements” of conformational propensities. In order to address this issue, He *et al.* recently measured the ^3^J(H^N^H^α^) constant and the CD spectra of various unblocked and blocked glycine based host-guest system for a representative set of host amino acid residues [139]. The authors found that four guest residues in GxG, AcGxGNH_2_, and AcGGxGGNH_2_, and the respective dipeptides exhibit slightly different ^3^J(H^N^H^α^) coupling constants at different pH. From this analysis the authors concluded that there is a notable influence of terminal groups. Using a two-state analysis of ^3^J(H^N^H^α^) coupling data at a single (room) temperature along with reference J_pPII_ and J_β_ values obtained from pPII/β maxima in coil libraries, they observed an increase in pPII content along the series (GxG) < (AcGxGNH_2_) < (AcGGxGGNH_2_). For example, it was found that the free terminal groups of GxG cause a 15% reduction of pPII propensities of the central residue, and hence blocked dipeptides or blocked glycine-based host-guest systems would be more appropriate model systems. However, caution has to be taken when analyzing ^3^J(H^N^H^α^)-constants since the observed differences between corresponding GxG, AcGxGNH_2_, and AcGGxGGNH_2_ coupling constants may also arise from small shifts of conformational distributions in the Ramachandran space.

**Figure 9:**
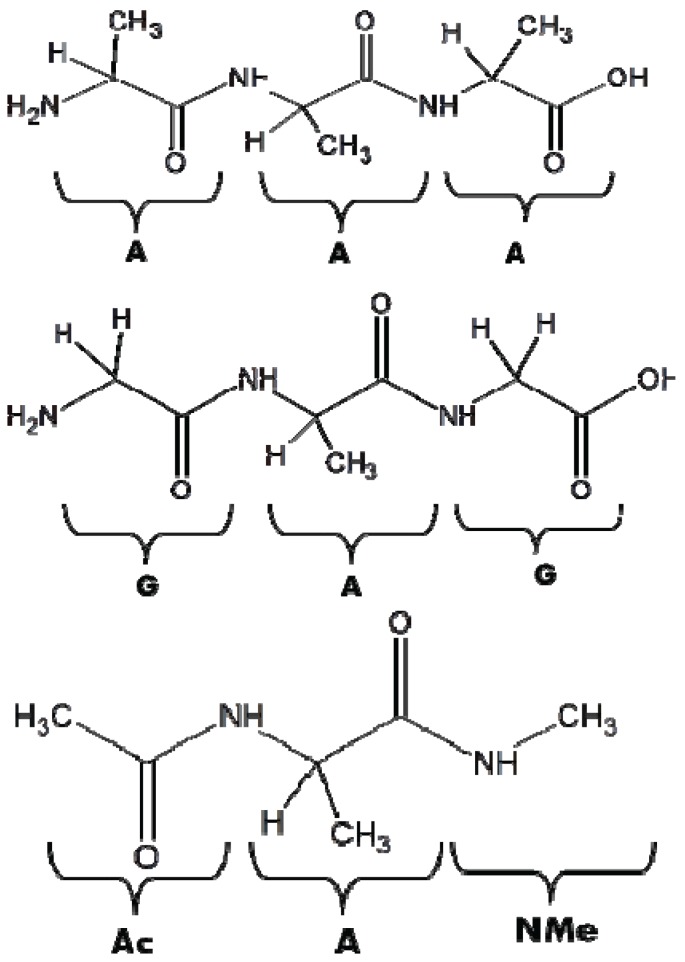
Unblocked AAA (**upper**), AdP (**middle**), and unlocked GAG peptide (**lower panel**).

To determine whether changing the protonation states of the N- and C-termini substantially influence the conformational manifold of the central amino acid residue in tripeptides, Toal *et al.* examined the pH-dependence of unblocked trialanine and the conformational preferences of alanine in the alanine dipeptide [29]. Earlier work on unblocked GAG was used for comparison (Figure 9 shows the structure of AAA, AdP and GAG for ref. [15,52]) Based on a global analysis of amide I’ band profiles and NMR and J-coupling constants the authors concluded that the conformational ensemble of trialanine as a whole, and the pPII content (χ_pPII_ = 0.84) in particular, remains nearly unaffected by changing the peptide’s protonation state. In addition, they found that the alanine dipeptide has slightly lower pPII content (χpPII = 0.74) and a conformational ensemble very similar to that of unblocked GAG model peptide, which indicates that both these peptides may be suitable model systems for studying the intrinsic conformational propensities. The minor conformational differences between AAA and AdP reflect the nearest neighbor interactions between alanine residues. This finding is of particular relevance for an experimental comparison and evaluation of results from MD studies, which are the subject of the next sub-chapter.

#### 2.2.2. Theoretical Studies on Alanine

Over the last fifty years the alanine dipeptide has been the classical model system for studying the conformational sampling of amino acid residues in the unfolded state. Numerous MD studies still use this peptide to explore the conformational propensity of alanine in water [15,68,125,126,127,128,129,142,143,145,146]. Most of the results of these and similar studies on other alanine based peptides [66,71,147] still predict statistical-coil like distributions for short alanine peptides and hence are at odds with the most of the aforementioned experimental results. This does not concern only the above described propensity studies but also simulations of, e.g., helix-coil transitions, for which many MD force fields just overestimate the nucleation parameter σ owing to their intrinsic oversampling of helical conformations. This tendency is kept alive even in some more recent MD simulations. For instance, Beck *et al.* reported “intrinsic” propensities of amino acids using *in lucem* MD simulations in the host-guest motif GGXGG and reported a significantly lower pPII propensity (16%) and an α-helical propensity above 50% for alanine [144]. Thus, they also actually predicted a departure from a local random coil distribution, but with a preference for right-handed helical rather than for pPII conformations. Generally, the results of MD simulations with regard to unfolded peptides depend heavily on the choice of the force field, as demonstrated with systematic comparisons by Zagrovic *et al.* [66] and Kwac *et al.* [143]. Among several attempts to move the results of MD simulations closer to experimental constraints, some success is noteworthy. In a computational study by Mu *et al.* in which the GROMOS package was utilized with Amber 09, it was also found that extended pPII and β conformations dominate for trialanine (65% and 12% respectively) [148]. Gnankaran and Garcia found that the Amber 96 force field could be forced to yield good agreement with experiment only by elimination of a backbone dihedral potential [149]. However, the physical rationale for these changes (*i.e.*, eliminating the torsional potential for φ and ψ in AMBER and a highly polarizable version of CHARMM) remains somewhat unclear. By adopting that strategy they were able to reproduce the very high pPII fraction of polyalanines. Moreover, Garcia found pPII to be particularly stable for tetralanine because four residues are necessary for an optimal backbone solvation in PpII [150]. In line with Garcia’s results, Kentsis *et al.* utilized ergodic sampling algorithms to show that in explicit water pPII is the predominant state of polyalanines [151]. Contrary, Sosnick and coworkers used many different force fields to calculate the MD populations for the central residue in trialanine and found a significantly reduced pPII propensity for most force fields, except for the OPLS-AA-97 force field, which reported >80% PPII propensity [152].

**Figure 10:**
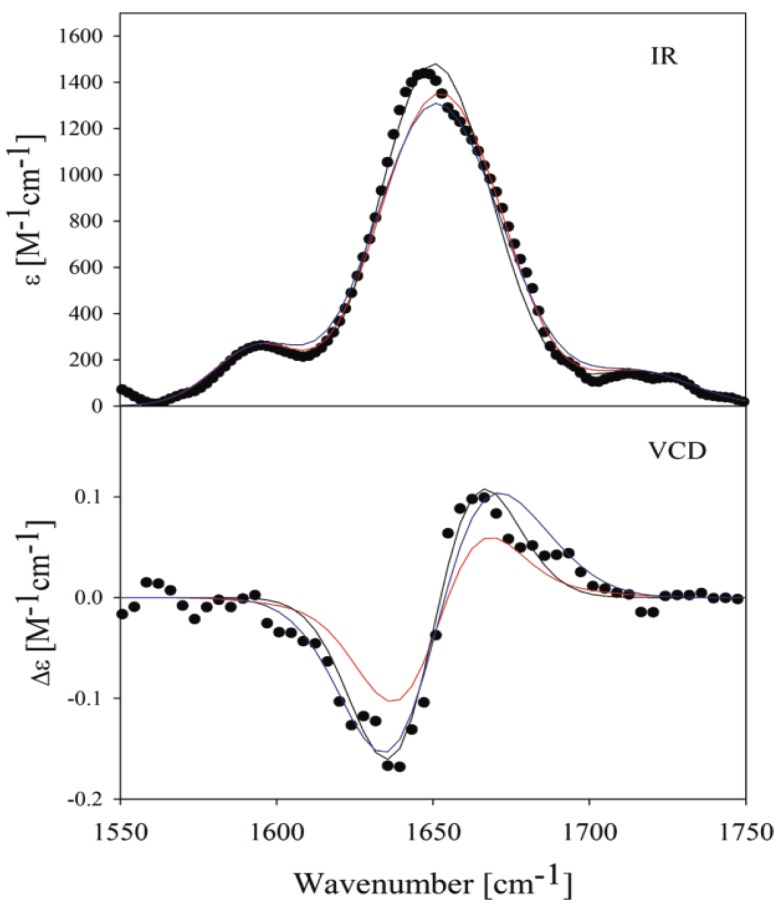
Amide I’ region of the infrared and vibrational circular dichroism spectra of A_5_W in D_2_O. The red lines result from a simulation using a conformational distribution reflecting the Ramachandran plot obtained from MD simulations with a ff03* force field. The black lines reflect the results of a fit with an adjustable conformational model describable as superposition of two-dimensional Gaussian distributions associated with pPII, β-strand, right-handed helical, and inverse γ-turn-like conformations. The blue line was computed with a refined model, which additionally considered a further modified distribution as mentioned in the results. (Taken from ref. [153] with permission).

More recently, Best and Hummer tried to remedy the confusing situation by using Garcia’s insights but avoiding the rather drastic change of AMBER force fields [71]. These authors modified rather than eliminated the dihedral backbone force constants in two different Amber force fields (ff03 and ff99SB) to reproduce experimental data on the fraction of helix measured in short peptides. In addition to re-parameterizing the force fields based on quantum chemical calculations, the respective force fields were optimized to account for helical content of short peptides by modifying the torsional dihedral angle functions. However, although these authors were able to mostly reproduce the J-coupling constants that Graf *et al.* [120] reported for polyalanines reasonably well, their distribution still yielded a much lower pPII content for the respective polyalanine (0.5 for ff03* and 0.4 for ff99SB*). To investigate this further, Verbaro *et al.* used the conformational distributions obtained by Best and Hummer to simulate the amide I’ band profile of the VCD and IR spectrum of the A_5_W peptide and found them to be incapable of reproducing the experimentally obtained strong amide I’ VCD couplet (Figure 10) which is a hallmark of peptides with a large pPII content [153]. Moreover, these distributions led to an overestimation of the end-to-end distance, which these authors assessed by fluorescence resonance energy transfer (FRET) measurements on fluorescently labeled A_5_W. A subsequent modification of the conformational distributions required a higher pPII fraction in order to reproduce all experimental data (Figure 10), including the end-to-end distance.

**Figure 11:**
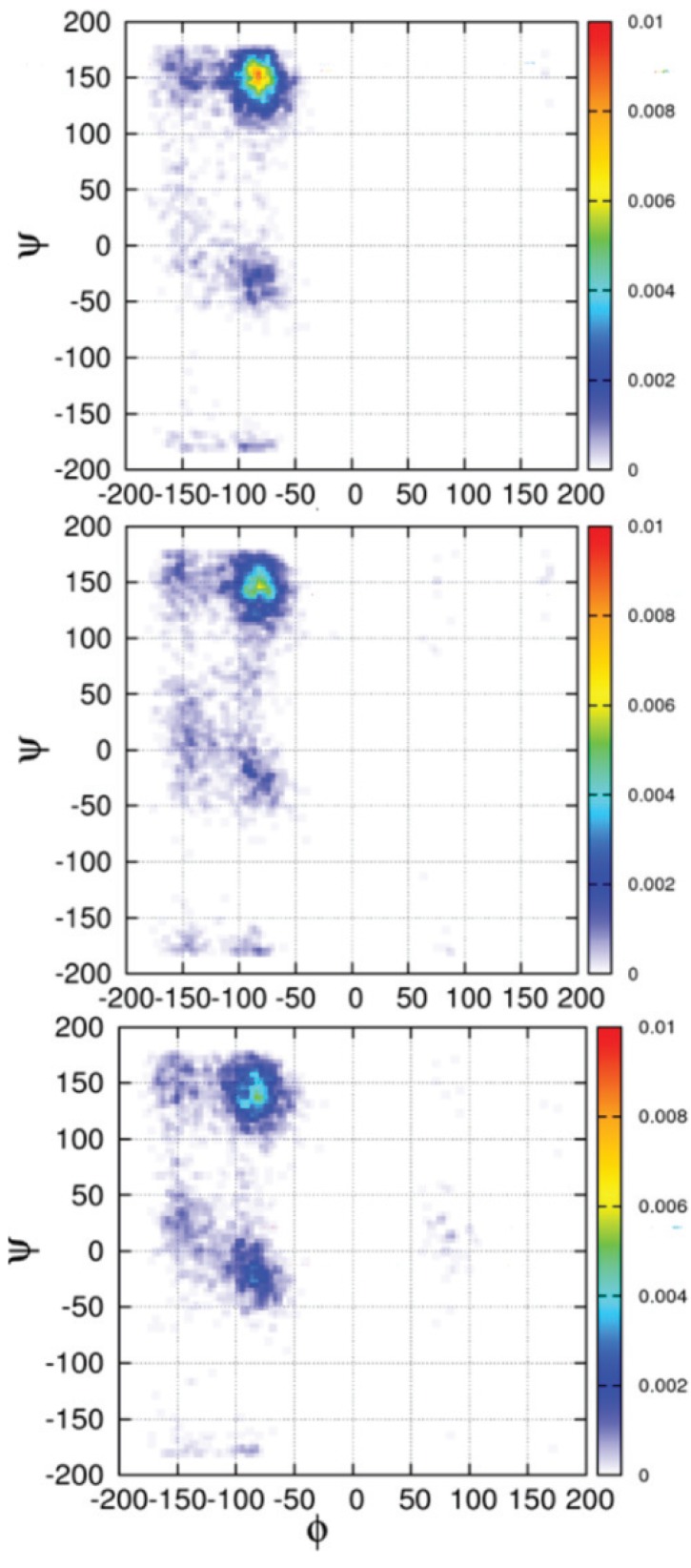
Ramachandran plots for cationic AAA (upper panel) and zwitterionic AAA (middle panel) and AdP (lower panel) obtained by MD simulations using the OPLS force field and SPC/E water model. (Taken from ref. [15] with permission).

Although much of the discrepancy with MD simulations of unfolded peptides likely resides in force field parameterization, some studies have indicated that the underlying reason might be more complex. In particular, the choice of water model was found to be important. Realizing the above shortcomings of MD simulations to reproduce many experimental observations for short peptides, Duan *et al.* used quantum mechanical methods with continuum solvent models and an effective dielectric constant (ε = 4) to account for the polarizability in the system [154]. The authors were thus able to determine that the pPII region is the most favorable. In a more recent approach by Lanza *et al.* [155] N-terminal blocked alanine peptides Ac-Ala-NH_2_ (N = 2–4) were studied using MP2, CCSD(T) and DFT *ab initio* methods with implicit hydration. These authors found that nearly all major conformations (alpha helical, pPII, β-strand, and turn-like conformations) as well as a large number of mixed structures are energetically accessible, more in agreement with statistical coil models. This result indirectly corroborates findings of Han *et al.* [121], who showed on a lower level of theory that obtaining a preference for pPII requires the explicit consideration of water. All these studies point to the role of hydration for conformational stabilization, and, with respect to theoretical calculations, the choice of water representation (*i.e.*, implicit *vs.* explicit solvation) and water model. Indeed, the water model seems particularly relevant for simulating unfolded states and intrinsically disordered peptides, for which energetic differences between conformations are generally on the order of RT and interactions with water are enhanced due to larger solvent exposure. The most commonly used water models for MD studies are the so called “three site” water models, namely, TIP3P, SPC, and SPCE, and the four site water model TIP4P [72,156,157,158]. Although these models are still widely used in the protein community, numerous studies have shown that the resulting conformational ensembles obtained by using these models are far from being in agreement with each other and with experimental findings. Hence, many attempts have been conducted to re-parameterize these water models. Notable in this regard is a study conducted by the Head-Gordon group in which they re-parameterized the standard TIP4P water model (TIP4P-ew) with inclusion of both Coulomb and Lennard-Jones long-range interactions [159]. The authors found that the TIP4P-Ew water model yielded slightly better agreement with experimentally measured scalar couplings for A_3_. However, they still obtained a conformational ensemble with under-estimated (relative to many experimental results) pPII fractions (pPII 52%, 40% β and 6% [160].) Using this water model Wickstrom *et al.* demonstrated that ensembles generated with TIP4P-Ew predicted the NMR scalar couplings for A_3_ reported by Graf *et al.* more accurately than ensembles generated with TIP3P model. In a recent study, Toal *et al.* [15] combined two common force fields (OPLS and Amber 03) with several commonly used water models (TIP3P, SPCE, TIP4P, and TIP4P-Ew) and found that the SPCE water model yielded the best agreement with experiment, that is, the greatest sampling of pPII conformations for cationic AAA and AdP. Figure 11 shows the conformational distributions these authors obtained using the OPLS/SPCE combination for the central alanine residue in for cationic AAA, zwitterionic AAA as well as AdP, which all show a remarkably similar conformational ensembles with pPII dominance.

In an effort to tackle the discrepancies between these water models, recently polarizable systems have been developed which, by modeling the solvent as a polarizable continuum, enhance the ability to reproduce water in different phases [161,162]. For instance, Kwac *et al.* performed MD simulations of AdP with several normal and polarizable force fields and different water models and found that only the combination of a polarizable AMBER ff02 force field with a polarizable water model yielded pPII fraction slightly higher than 0.5 [143]. However, at this time, polarizable model systems are still too computationally expensive to be widely viable for routine conformational studies.

## 3. Conformational Biases of Other Amino Acid Residues

### 3.1. Experimental Studies

**Figure 12:**
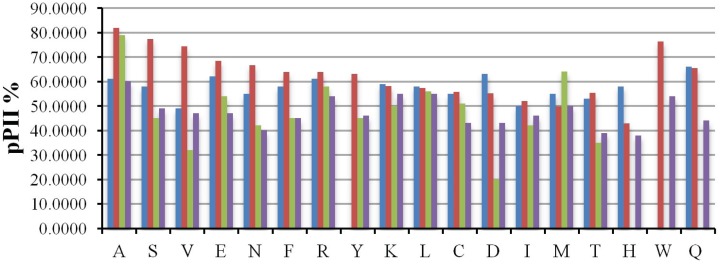
Comparison of (**a**) pPII; and (**b**) β-strand populations for guest residues as obtained by Rucker *et al.* for PxP (blue) [163], Shi *et al.* for GGxGG (red) [51], Hagarman *et al.* for GxG (green) [52,77,138], and Grdadolnik *et al.* [76] for XdP (purple).

**Figure 13:**
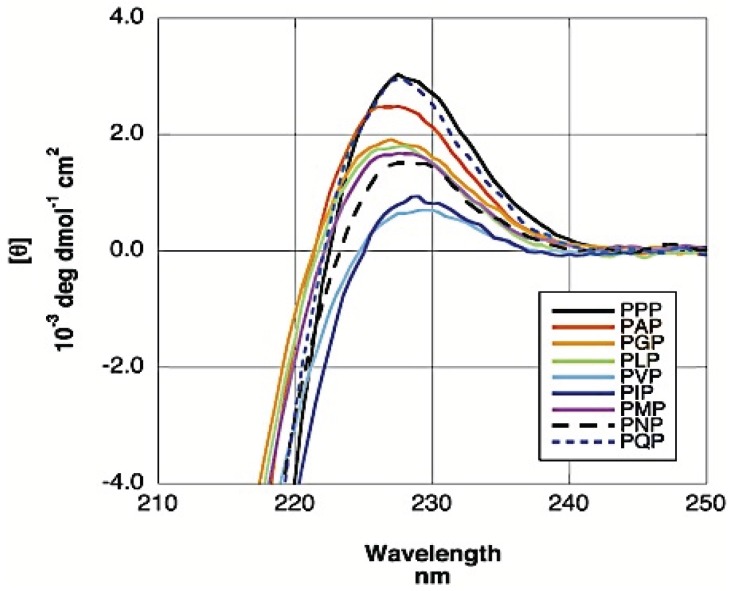
UV-CD spectra around the maximum dichroism value for PPP, PAP, PGP, PLP, PMP, PIP, PVP, PNP, PQP. (Taken from ref. [163] with permission).

While experimental results have generally converged in measuring a high pPII propensity for alanine, conformational propensity studies on residues other than alanine are more limited in number and vary in terms of the choice of short peptide model system. Figure 12 displays the amino acid propensities obtained utilizing various model systems, which will be discussed in detail below. One of the first studies in this regard was conducted by Creamer and colleagues who explored the residue level bias for pPII by studying short polyproline based peptides, PPP-X-PPPGY where x = A, G, V, L, I, N, Q, and M, with UVCD spectroscopy [163,164]. The spectra for all six peptides at the characteristic maximal dichroism for pPII (θ_228 nm_ for polyprolines)) is re-produced in Figure 13.

By assigning the ellipticity value displayed in the UVCD spectrum of poly-L-proline in 8.4 M GuCl (=7600 deg dmol^−1^∙cm^2^) at 228 nm to 100% and the *θ_max_* value of −4300 deg dmol^−1^∙cm^2^ to a completely disordered peptide, they derived the following equation to estimate the pPII percentage of their peptides:

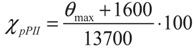
(2)
where *θ_max_* is the experimentally obtained molar ellipticity at the characteristic pPII maximum wavelength of 228 nm. They found that proline has the highest pPII content followed by glutamine and alanine. In general, the hierarchy of pPII preference obtained follows the intensity of the maximal dichroism shown in Figure 12. The lowest pPII propensities were found for branched amino acid residues (I, V) (Figure 13). Kelly *et al.* postulated that the residue specific range in these conformational propensities reflect the accessibility of the guest residues’ backbone for solvent interaction [164].

The above analysis of Creamer and associates was recently challenged on computational ground by Moradi *et al.* [165] The authors obtained the conformational sampling of different proline and non-proline amino acid residues in blocked P_3_XP_3_GY peptides with the Amberff99SB force field. They found that the conformational distribution of the proline residue preceding the guest residue X depends heavily on the latter, which is indicative of strong nearest neighbor interactions. All host residues besides P, Y and W were found to increase the *trans-*population of the preceding proline and its sampling of right-handed helix like conformations. With respect to the conformational distribution of the investigated X-residues the authors obtained a dominant sampling of the upper left quadrant of the Ramachandran plot, but all residues show a clear preference of a β-strand like conformation over pPII. Thus, the contribution of x to the overall pPII content of the peptide was found to be modest. The authors lend credibility to their study by showing that the Gibbs energy of PX nearest neighbor interaction that they obtained from an odds-ratio analysis correlates with the overall pPII content that Rucker *et al.* derived from their UVCD data [163]. In a subsequent paper Moradi *et al.* made similar observation for P_3_x_n_P_3_GY peptides [166]. They found that only the X-residue preceding the C-terminal proline sequence exhibit a significant pPII fraction, whereas all residues including alanine which are located in the center of the X_n_-sequences exhibit rather low pPII propensities. The authors concluded that non-proline residues do not have a propensity for pPII. We wonder whether the way in which Rucker *et al.* [163] analyzed their CD spectra is physically justified. It is very much based on the assumption that all amino acid residues exhibit the same Δε value at the positive maximum of the UVCD spectrum, if they adopt a pPII like conformation. This assumption would only be justified if the position and width for pPII sub-distributions were the same for all amino acid residues. The work of Hagarman *et al.* as well as coil library distributions show that this is definitely not the case [52,77,79,81]. Corroborating their results Toal *et al.* recently reported rather substantial differences between Δε_pPII_ values for the positive CD maxima of different amino acid residues in GxG peptide [167]. These results again reveal the rather substantial differences between the results of simulated and experimentally conformational propensities.

Eker *et al.* chose an alanine based host-guest system, AXA, where x = G, V, M, H, S, P, L, K, Y, and F, to experimentally investigate the conformation of guest residues in aqueous solution [119]. They combined Fourier transform IR, polarized Raman spectroscopy, and vibrational CD measurements of the amide I’ band profile of alanyl-X-alanine tripeptides in D_2_O to obtain the dihedral angles of a representative conformation of the central amino acid residue, as outlined above for AAA. They checked their results qualitatively by measuring the respective UVCD spectra as well. The obtained results led them to sort the investigated peptides into three classes. Valine, phenylalanine, tryptophan, histidine, and serine predominantly adopt an extended β-strand conformation. Cationic lysine and proline prefer a polyproline II-like structure. The authors reported that alanine, methionine, glycine, and leucine populate these two conformations with comparable probability.

In attempts to determine the fractions of each sub-population reflecting the intrinsic conformational propensity of amino acid residues, many researchers have turned to glycine based model systems. Glycine based host-guest systems had been frequently used as models beforehand to obtain ^3^J(H^N^H^α^) and chemical shift values for what researchers considered as “random coil” conformations of amino acid residues [168,169,170,171,172]. One of the first conformational propensity studies in this regard was conducted by Shi *et al.* [51,137], who chose Ac-GGXGG-NH_2_ in water, where x represents the 19 natural amino acids except glycine, to investigate the guest residues conformational preference in the unfolded state. Glycine as neighbor is considered as an ideal reference residue as it ensures minimal nearest neighbor interaction due to its hydrogen based side chain, thus allowing for an accurate intrinsic propensity scale. Moreover, since glycine is non-chiral, CD spectra can be expected to reflect solely the conformational distribution of the X-residue. Shi *et al.* measured the UVCD spectra for 18 residues (glycine was excluded, alanine had been measured previously) at three different temperatures. For most of the investigated peptides the spectra exhibit isodichroic points, indicating a two-state model, which was assigned to a pPII/β equilibrium. To obtain quantitative information on the amount of pPII/β present, the authors utilized the conformational sensitive coupling constants derived from H NMR experiments, the relationship of which to φ is discussed above (equation (1)) and visualized in Figure 7. As shown by the respective Karplus plot in this figure, the ^3^J(H^N^H^α^) coupling constant varies dramatically in the φ region between pPII and β strand (−65°–125°, respectively). Thus, ^3^J(H^N^H^α^) provides a rather sensitive measure of the angle φ. Assuming that each peptide exists in either pPII or β strand conformation, the authors calculated the pPII percentage from the experimental ^3^J(H^N^H^α^) as follows:

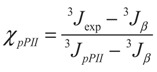
(3)
where *J_ppII_* and *J_β_* are reference ^3^*J* coupling constants obtained from the maxima of the respective subdistributions in the coil libraries of Avbelj and Baldwin [73,85]. They represent the maxima of respective residue distributions in the pPII and β-strand region of the Ramachandran plot, respectively. ^3^*J*_exp_ denotes the experimental J-coupling constant. The authors reported their results of H-NMR and UVCD analysis of these peptides as indicating high proportions of pPII conformations at low temperature for nearly all investigated residues, shifting to an increasing β-strand population at high temperature for all peptides. The extent of pPII preference they obtained differs with each host residue underscoring the idea that each amino acid indeed has distinct conformational preference in the unfolded state. They are shown for reference in Figure 13. The pPII content thus obtained ranged from 40% to 80% depending on the residue, with alanine, not surprisingly, exhibiting the highest pPII propensity of 83%.

The results reported by Shi *et al.* [51] have been challenged on various accounts. It has been argued that their analysis of experimental ^3^*J* coupling is questionable, because (1) the ^3^*J*(T) coupling constants should represent Boltzmann averaged conformational distributions rather than just two isolated conformations, (2) experimental ^3^*J* coupling constants are generally three-fold degenerate with respect to the angle φ and hence could be reproduced with various conformational ensembles [69,70] and (3) the reference ^3^*J* coupling constants used to represent pure *J_pPII_* and *J_β_* in these studies were obtained from coil libraries [71] which may not be actually reflect real distributions of amino acid residues in unfolded peptides. In this context one might also question whether the utilization of an unrestricted coil library, which exhibits quite substantial a right-handed helical fraction, is a suitable choice for a two-state analysis that considers only a pPII-β-strand equilibrium. Regardless of this debate, the results of Shi *et al.* can be considered as underscoring the notion that different amino acid side-chains implement distinct conformational propensity in the unfolded state.

**Figure 14:**
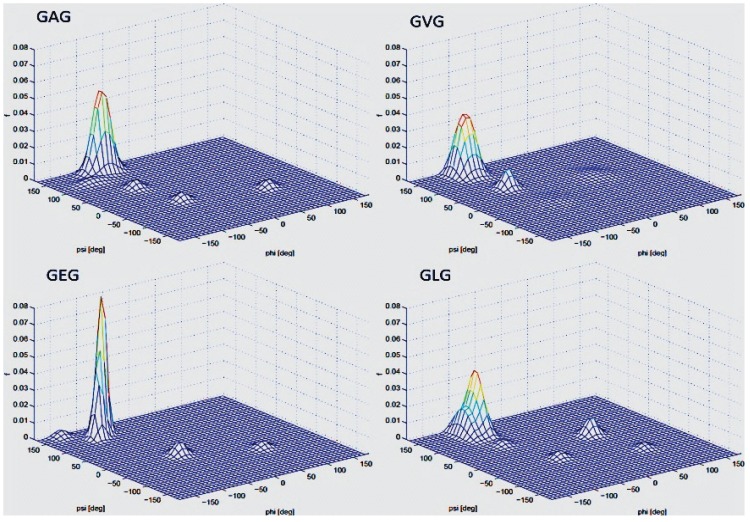
Conformational distributions of the central residue in GAG, GVG, GLG, and GEG obtained from analysis of amide I’ band profiles and J coupling constants, illustrating the 2D distribution approach used by Hagarman *et al.* (Taken from ref. [52] with permission).

A detailed conformational analysis aimed at obtaining realistic conformational ensembles of amino acid residues was performed by Hagarman *et al.* [52,77,78,138] using unblocked GxG as host-guest system. As previously mentioned, these authors described individual conformational distributions of the guest residue by constructing variable two-dimensional Gaussian functions in the φ,ψ space, and hence, they tried to avoid using less realistic representative structures. Using these functions in the analysis of amide I’ band profiles and various J-coupling constants, they were able to obtain Ramachandran plots for 15 amino acid residues. A representative set is shown in Figure 13. The authors confirm the high intrinisic pPII propensity of alanine (79%) as well as the variable conformational preferences of all other residues. However, the authors report that alanine is the only residue with such a high pPII fraction. The conformational ensemble obtained for alanine in GxG is shown in Figure 14 for reference

**Figure 15:**
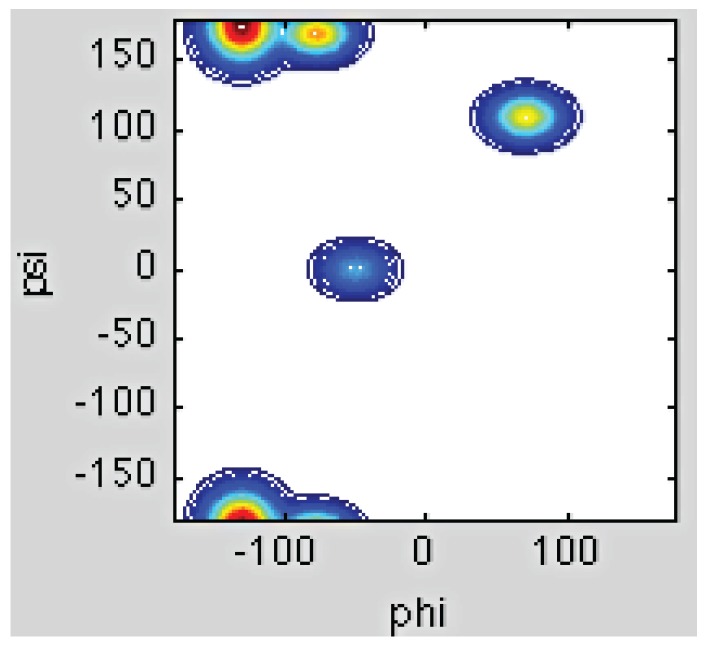
Conformation distribution of the central residue in unblocked GDG illustrating the high turn propensities of aspartic acid. Taken from [78] and modified.

In general, the pPII scale derived by Hagarman reads as follows: A, M > L, E, R, K>>I, V, S > D, N, T, C, with most residues exhibiting a lesser pPII content than the corresponding values reported by Shi *et al.* [51] Generally, the ensembles that these authors obtain show that most residues are dominated by combined pPII and β-strand sampling (>80%) (Figure 13). The remaining fraction is distributed over different types of turn-like conformations; right-handed helical sampling is comparatively weak. However, it is noteworthy that Hagarman *et al.* and Rybka *et al.* surprisingly found that individual turn-like conformations, some of which are supported by intra-peptide H-bonding, may constitute up to 23% of the intrinsic conformational ensemble of a residue [77,78]. Figure 15 shows the conformation distribution these authors obtained for protonated aspartic acid, which displays an unexpected and relatively high preferences for so called asx-turns, which are supported by intra-peptide H-bonding between the C=O group of the side chain and the C-terminal amide proton. Aspartic acid, threonine, and asparagine are most notable in his regard with unusually large >20% turn populations, which have more recently been confirmed with temperature dependent 2D NMR studies.

**Figure 16:**
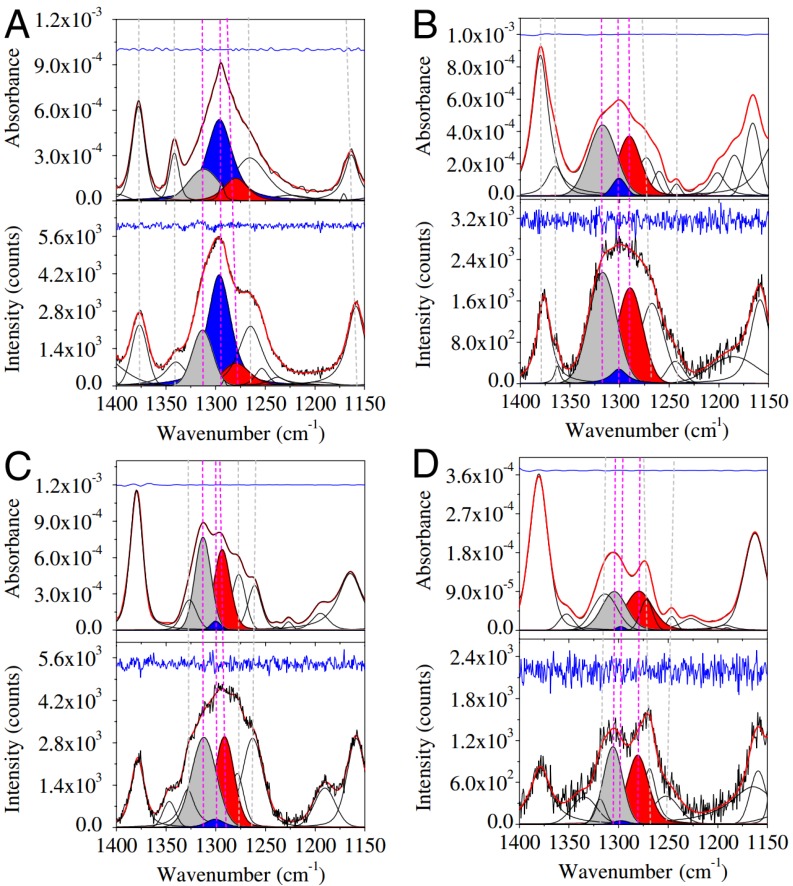
Experimental (black line) and fitted (red line) ATR-absorbance (**Upper**) and Raman (**Lower**) spectra (amide III region) for four dipeptides: (**A**), glycine; (**B**), arginine; (**C**), methionine; and (**D**), isoleucine. The three colored components are gray (band A) PII; blue (band B) αR; red (band C) β. Taken from ref. [76] with permission.

More recently, Grdadolnik *et al.* [76] performed a comprehensive experimental study and reported propensity scales for 19 non-proline residues in blocked dipeptides (*i.e.*, XdP) based on an analysis of the amide III’ region of their Raman and IR spectra as well as the φ-dependent ^3^*J* coupling constant [76]. By measuring the amide III’ region with both attenuated total reflection IR spectroscopy and Raman spectroscopy, they assigned three resolvable sub-bands to pPII, β, or right-handed helical conformers (Figure 16). By considering these three main conformations they were able to obtain pPII fractions of 0.68 and 0.53 and β fractions of 0.17 and 0.43 for alanine and valine, respectively, more in line with the results of Hagarman *et al.* and Schweitzer-Stenner *et al.* [52,138]. These conformations distributions are also compared in Figure 13 for reference.

### 3.2. Analysis of Coil Libraries

**Figure 17:**
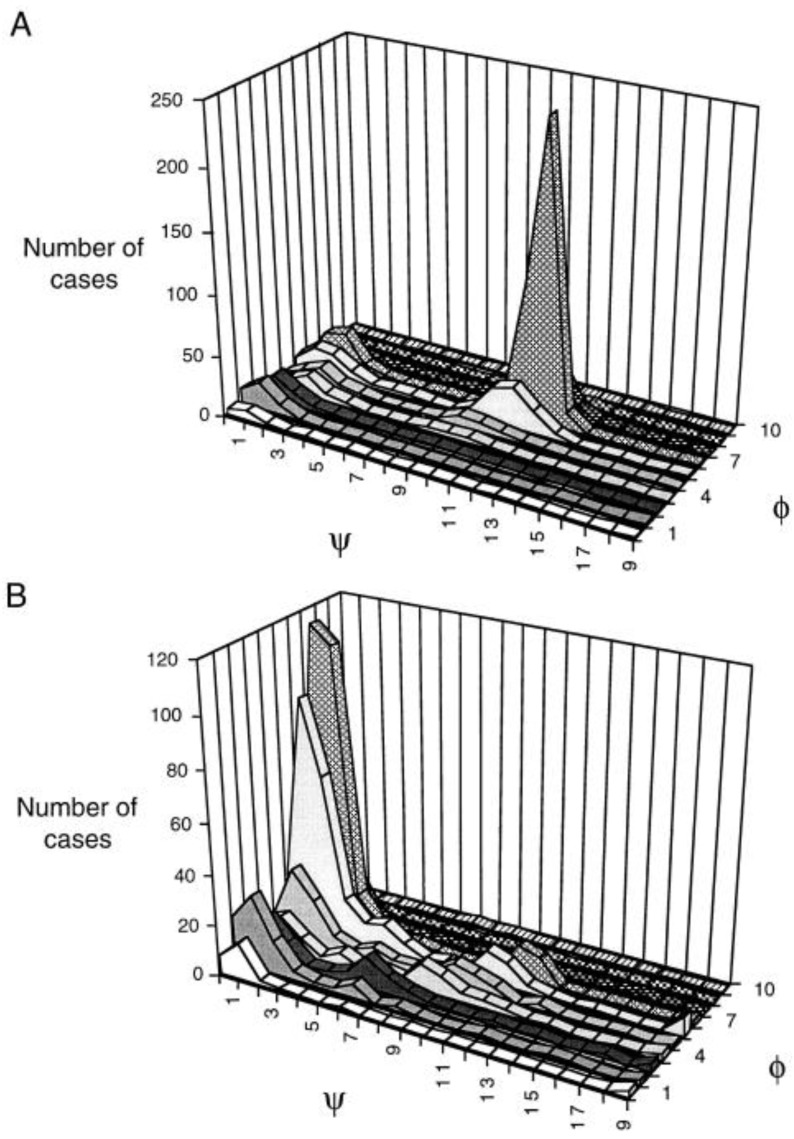
Distribution obtained in the upper left quadrant of the Ramachandran plot for Alanine using (**A**) all secondary structure conformations in the protein database (*i.e.*, an unrestricted library) or (**B**) only those alanine residues in a coil conformations (*i.e.*, helices, sheets, turns omitted). The number of cases was normalized values to 1000. Each axis marker represents an 18° interval. Taken from ref. [75] with permission.

An alternative strategy for determining the conformational preferences of amino acids in the unfolded state is the analysis of conformational distributions found in coil libraries. In general, there are two main types of coil libraries constructed for structure prediction. So called “unrestricted” coil libraries simply consider large sets of proteins from the Protein Data Bank (PDB) without any resections as to the secondary structure elements of selected proteins. This strategy was based on the argument that the contextual influence would be eliminated if one averages over many protein environments [37]. Support for this assumption is based on the linear correlations between average ^3^J(H^N^H^α^) coupling constants derived from unrestricted libraries and corresponding values obtained for AcGGxGGNH2 peptides in water. However, distributions obtained from these types of libraries are still noticeably biased towards right-hand helical conformations and in general, do not agree with experimentally derived distributions of amino acids in solution [37,75,81]. In contrast to the unrestricted library, only a subset of the database can be chosen in which certain types of secondary structure sequences are purposefully omitted. This way, any possible effect of the secondary structure on the amino acid conformation is effectively eliminated hence mimicking the unfolded state. Swindells *et al.* constructed a restricted “coil library” along this line by considering only residues in coil regions and omitting residues lying within alpha helical or beta strand structures within a dataset of 85 proteins from the PDB [173]. By using this library they determined that correlations between intrinsic conformational propensities and observed secondary structure propensities for helices are modest and strongest for β strand propensities/structures. Serrano took this notion a step further by constructing a coil library in which all regular secondary structures were omitted, including amino acids in β-turns, which could also have associated nonlocal interactions [75]. As displayed in Figure 17, the author was able to show that the distribution for alanine dramatically changes from mostly right-handed helical to mostly pPII when removing all secondary structure conformations (including β turn conformations), a finding being at least in qualitative agreement with most experimental studies on alanine. However, as noted by Jha *et al.* [80], these restricted coil libraries may now exhibit a systematic bias towards the pPII conformation due to their inclusion of residues at the ends of structured regions, which would inherently disfavor sheets and helices (and hence favor pPII conformations).

**Figure 18:**
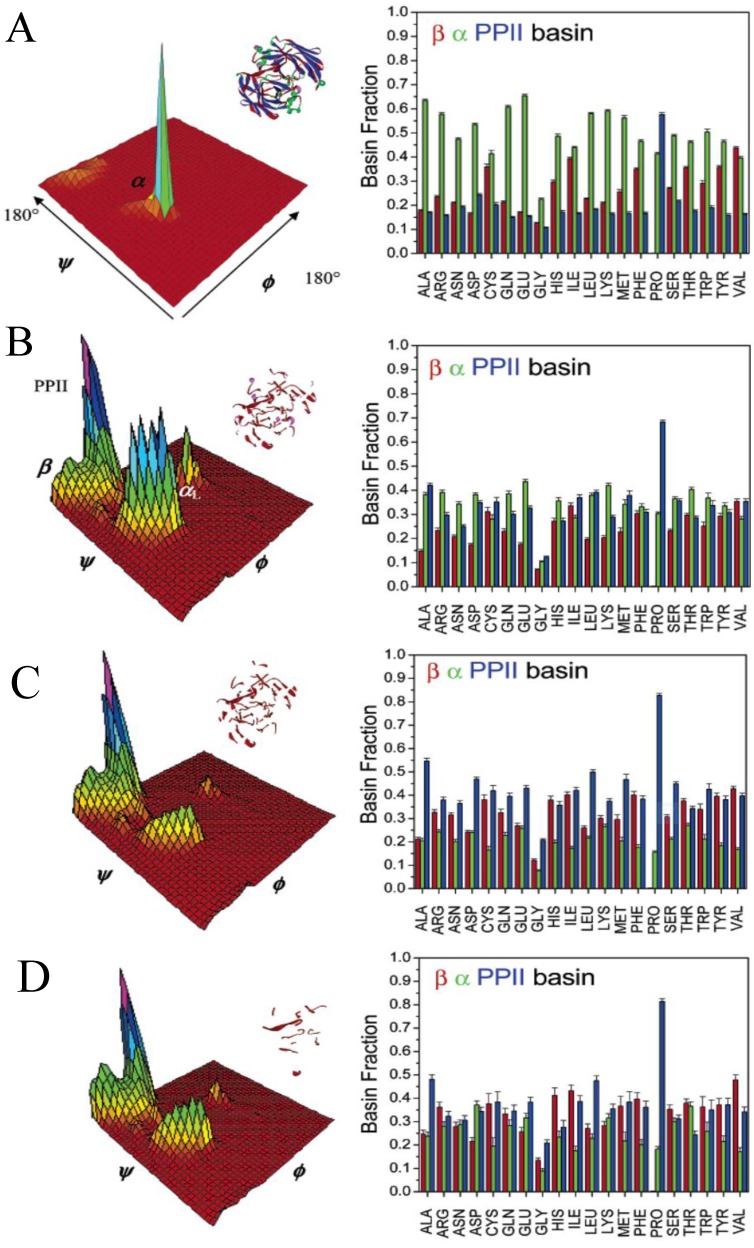
Basin preferences in different coil libraries. Probability distribution in the Ramachandran plane of all residues (except Gly and Pro) for the (**A**) entire PDB, (**B**) library without helices and sheets (**C**) library without helices, sheets, and turns, and (**D**) library without helices, sheets, turns, and terminal, pre-proline, and most exposed residues. Basin fractions for the 20 amino acids are shown in adjacent panels for the corresponding libraries described in panels **A**–**D**. (Taken from ref. [81] and modified).

These authors constructed a much more restricted coil library in which they tried to eliminate this bias by omitted residues flanking regular secondary structures (including prolines) and considering only residues that lie in “coil” stretches of four or more. Figure 18 displays the (φ,ψ) basin preferences from each of the aforementioned libraries as derived by Jha *et al.* [81] The respective plots clearly shows that there is still a moderate preference for the pPII conformation in the most restricted coil library (library without helices, sheets, turns, and terminal, pre-proline, and most exposed residues) for most residues, with the highest pPII preference seen for alanine (49%). High pPII levels were found even for buried residues indicating that preferential solvation may not be the only contributor to pPII preference in the unfolded state.

The question arises whether the distributions obtained from coil libraries reflect the respective intrinsic propensities in the unfolded states for individual amino acid residues. Jha *et al.* [81] compared the pPII preferences of their coil library studies to those experimentally obtained by Shi *et al.* for GGxGG peptides and found only a modest correlation (*R* = 0.6) [51]. However, this comparison may be problematic as the coil library averages over all neighboring amino acid residues, which may not completely eliminate specific nearest-neighbor interactions. Schweitzer-Stenner *et al.* specifically compared the conformational distributions of GYG, GFG, GIG, GVG, GRG, and GEG tripeptides in water with those obtained for the same tripeptide sequences from a restricted (helices and sheets omitted) coil library [138]. As shown in Table 1, the coil library distributions in general have a much larger fraction of type II’/type III β turn population (close to the trough of right handed helical conformations in the Ramachandran plot), indicating that even amino acid residues in coil regions experience non-local interactions which shift their distribution towards turns.

**Table 1:** Comparison of Conformational Distributions of the Indicated GxG Peptides in Solution (Water) and in Truncated Coil Libraries [174]. Subset “c” represents those obtained coil libraries, subset “e” represents experimentally obtained distributions.

	pPII	β	II’/III turn	γ-turn	III’ turn	I’/γ turn
GYGc	0.38	0.19	0.31		0.05	
GYGe	0.45	0.34	0.16	0.12		
GFGc	0.29	0.21	0.39		0.1	
GFGe	0.45	0.45	0.05		0.05	
GIGc	0.26	0.36	0.38			
GIGe	0.42	0.37	0.18		0.08	
GVGc	0.33	0.28	0.39			
GVGe	0.32	0.46	0.04	0.11	0.07	
GRGc	0.31	0.15	0.48			0.06
GRGe	0.58	0.2	0.08	0.03	0.03	0.08
GEGc	0.39	0.17	0.41		0.03	
GEGe	0.54	0.32	0.08	0.04	0.04	

Interestingly, the authors found that the increase in these turn populations in coil library distributions is concomitant with rather asymmetric changes of the pPII/β-strand population. The gain in turns occurs predominantly at the expense of β-strand for Y, V and to a limited extent also for F and E, the gain in turns occurs mostly at the expense of β-strand, whereas pPII is the main source for R and F. These uncorrelated changes in β and pPII sampling imply that intraprotein interactions do not solely stabilize turn/helix conformations; in addition, they selectively destabilize either pPII or β-strand conformations. The authors concluded that the assumption that coil libraries constitute a real ergodic canonical ensemble reminiscent of statistical coil may not be wholly true due to non-local interactions and that instead these libraries constitute an ensemble of polypeptides subjected to a statistical distribution of non-local interaction energies.

## 4. Nearest Neighbor Influence of Conformational Propensities

One of the pillars of Flory’s classical random coil model for the unfolded state of proteins is the isolated pair hypothesis (IPH) [12]. It stipulates that the conformational distribution of distinct residue in the polypeptide chain is totally independent from the nature and the adopted conformation of the two adjacent residues. As a consequence, a random coil chain carries a significant amount of conformational and combinatorial entropy, which more than 40 years ago led Levinthal to propose his paradoxon [8]. The total conformational entropy and energy can just be written as a sum of residue enthalpies and entropies. Since the conformational distribution of different amino acid residues were expected to be comparable (with the exception of proline and glycine), enthalpy and entropy would linearly increase with the number of residues.

Over the last ten years the validity of the IPH has been questioned based on experimental, computational and bioanalytical results. One of the first reports of deviations from IHP based expectations came from the analysis of ^3^J(H^N^Hα) constants of a 130-residue fragment of the unfolded fibronectin-binding protein from Staphylococcus aureus, a protein with three homologous and a terminal segment. In this study, Penkett *et al.* [84] showed that the average ^3^J(H^N^H^α^) constants of individual amino acid residues in this protein correlate well with expectation values derived from φ-distributions of coil libraries. However, a more detailed analysis revealed that individual ^3^J(H^N^H^α^) coupling constants depend on the nature of the respective neighbors. With respect to the strength of this nearest neighbor interaction, Penkett *et al.* divided the investigated residues into two categories: category L contained aromatic and branched aliphatic side chains (V, I, F, W, Y) and category S, which contain all the other residues with the exception of glycine. Category L residues were found to increase the ^3^J(H^N^H^α^) coupling constant of their downstream neighbors (0.4 Hz on average), whereas S-residues have a negligible influence. The authors interpreted the influence of L-residues as an equilibrium shift from α-helical to β-strand conformation. They invoked steric effects as a reason: extended β-strand conformations exhibits less steric strain than right-handed helices. The obtained ^3^J(H^N^H^α^) shifts shed some light of the origin of downfield shifts of ^15^N resonances of residues in the presence of upstream L-category neighbors, which were consequently also interpreted as resulting from nearest neighbor interactions.

**Figure 19:**
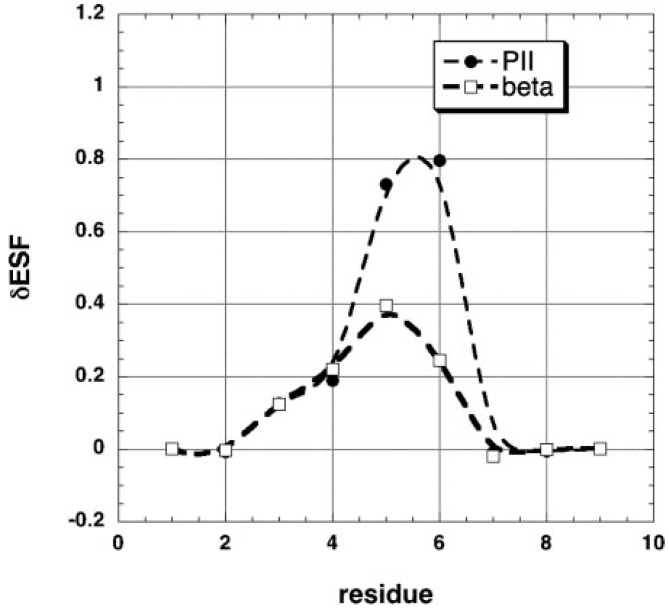
Representation of the change of the electrostatic solvation free energy induced by substituting the fifth alanine residue of a hepta-alanine peptide by valine. Changes are plotted for pPII and β-strand conformations, as indicated. (Taken from ref. [85] with permission).

Avbelj and Baldwin subjected the findings of Penkett *et al.* to a computational thermodynamic analysis [85]. The result of their study suggests that nearest neighbor interactions are dictated by solvent mediated processes rather than by direct steric interactions. Contrary to Penkett *et al.* they took pPII as a distinct residue conformation into consideration. They discussed three contributions to the φ-dependence of the overall Gibbs energy: (a) the torsional potential V(φ), (b) electrostatic interactions and (c) protein/peptide solvation and its modification by side chains. V(φ) exhibits a maximum (*i.e.*, its most destabilizing effect) at 120°; thus, it does not favor β-strand conformations. On the contrary, electrostatic interaction favor β-strand like conformations, but solvation screens Coulomb interactions thus allowing a dominance of V(φ), which in turn favors pPII like conformations. If side chains (like valine) perturb the hydration shell, they reduce the pPII propensity. The influence of side chains on propensities is not limited to their own residue. As discussed by Avbelj and Baldwin, side chains are capable of affecting the solvation of nearest neighbors as demonstrated in Figure 19, which shows how the substitution of the fifth residue of a hepta-alanine peptide by the β-branched valine increases the solvation Gibbs energy both in the pPII and the β-strand conformation. Increase here means less solvation since the solvation Gibbs energy is negative. The effect is much more pronounced for pPII than for β, thus causing a preferential de-stabilization of the former. These changes clearly affect the solvation of the neighbors, where it also favors the β-strand conformation. By performing similar calculations for other amino acids, Avbelj and Baldwin found that residues with branched residues and aromatic side chains exhibit a much stronger nearest neighbor effect, in qualitative accordance with Penkett *et al.* [84].

The role of the solvent was also emphasized in studies using the Hamiltonian replica exchange approach for a series of peptides with 2–5 peptide units [175]. In agreement with Avbelj and Baldwin the authors of this study found that valine as N-terminal neighbor of alanine stabilizes β-strand. However, a C-terminal neighbor valine actually stabilizes pPII even further. That is a very surprising result, and actually contradicts Avbelj and Baldwin, as clearly visible in Figure 19. Further experiments that quantify nearest-neighbor interactions are needed to clarify which of these simulations describe reality.

**Figure 20:**
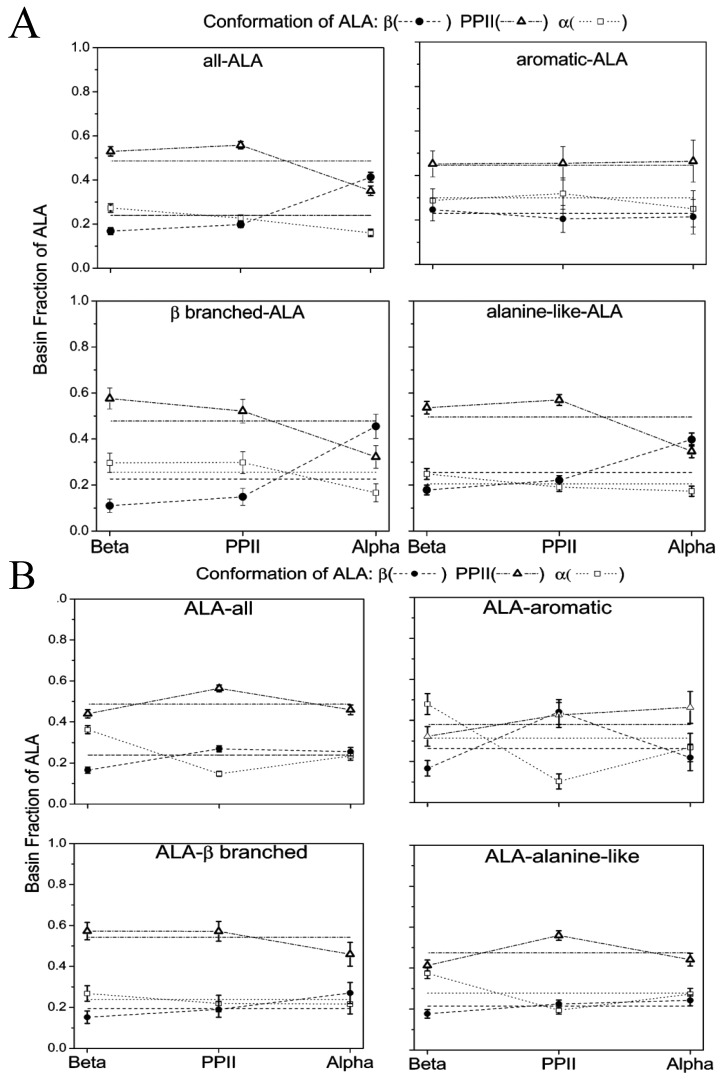
Representation of the change of the electrostatic solvation free energy induced by substituting the fifth alanine residue of a hepta-alanine peptide by valine. Changes are plotted for pPII and β-strand conformations, as indicated. Taken form ref. [81] and modified.

Thus far the most detailed information about nearest neighbor interactions came from coil library studies of Sosnick and coworkers, which were already discussed in detail in the preceding chapter. Jha *et al.* observed (a) that the contributions of nearest neighbors to apparent propensities of an amino acid residue can be significant, (b) that these interactions are both side chain and conformation dependent and (c) that they affect the propensities as well as the position of local maxima of distributions [81]. In order to simplify the representation of nearest neighbor effects, they classified neighbors according to the properties of their side chains: β-branched (and aliphatic), aromatic and alanine like. G and P were not considered. The influence of these different types of amino acid residues on the pPII, β-strand and right-handed helix population of alanine is visualized in Figure 20. Let’s start with the influence of the N-terminal neighbor. If it is β-branched (V, I), the pPII and β-fractions are actually slightly above the average value in coil libraries, but once the neighbor adopts a helical conformation, pPII drops and β-strand increases. Aromatic neighbors have very limited influence on the distribution, while alanine like neighbors behave just like valine and isoleucine. The situation is different for the C-terminal neighbor. An aromatic neighbor in pPII increases the β-strand population and decreases the helical content compared with β-strand and right handed helical. The pPII fraction of alanine is less affected, it is highest for a helical neighbor and lowest if the neighbor adopts β-strand. The influence of C-terminal β-branched neighbors is modest, but qualitatively similar to that of the corresponding N-terminal neighbor. Alanine-like N-terminal neighbors stabilize pPII and destabilize the helical conformation [15].

The influence of the residue conformation on nearest neighbor interactions was also investigated by Pappu *et al.* [82]. They used a rather simple hard-sphere model to explore the conformational space of a polyalanine peptide. The repulsive potential utilized in this model was augmented by hydrogen bonding formation for certain φ,ψ conformations. The Ramachandran space was subdivided into 49 quadratic meso-states and their population was calculated with Monte–Carlo methods. The authors thus found that steric clashes involving conformations in the right handed helical region of the Ramachandran plot (which violates the IPH), while no such clashes were obtained when neighbors adopted conformations in the upper left quadrant of the Ramachandran plot. Hence, this work questions the validity of the IPH, but only for a limited set of conformations. This is a somewhat less complex picture than that arising from the coil library studies of Sosnick and colleagues, even though the latter also suggest a strong nearest neighbor effect involving residues in helical conformations [81,83].

The significance of the deviation from IPH predictions obtained by Pappu *et al.* was subsequently questioned by Ohkubo and Brooks [176]. They used a CHARMM/GB force field to simulate the helix-coil transition of a polyalanine peptide. They found that the Zimm-Bragg parameters [177] s(T) and σ(T) “remain unchanged along the length of the peptide” unless very short chains were considered. The authors were also able to calculate the conformational entropy, and found it to increase linearly with the chain length. Based on these results, the authors reinstated the validity of the IHP.

A different message emerged from other studies of the Sosnick laboratory by which they tried to reproduce residual dipole coupling values of unfolded proteins observed with NMR experiments. To this end, Sosnick and coworkers examined coil library distributions of amino acid residues with and without nearest neighbor influences [83]. Considering the latter led to a much better reproduction of experimentally obtained coupling values, the results emphasized the relevance of these nearest neighbor interactions for the structural ensemble of unfolded proteins and peptides. In this context, Baxa *et al.* considered nearest neighbor interactions in order to estimate the loss of conformational entropy by the folding of a denatured ubiquitin [178].

**Figure 21:**
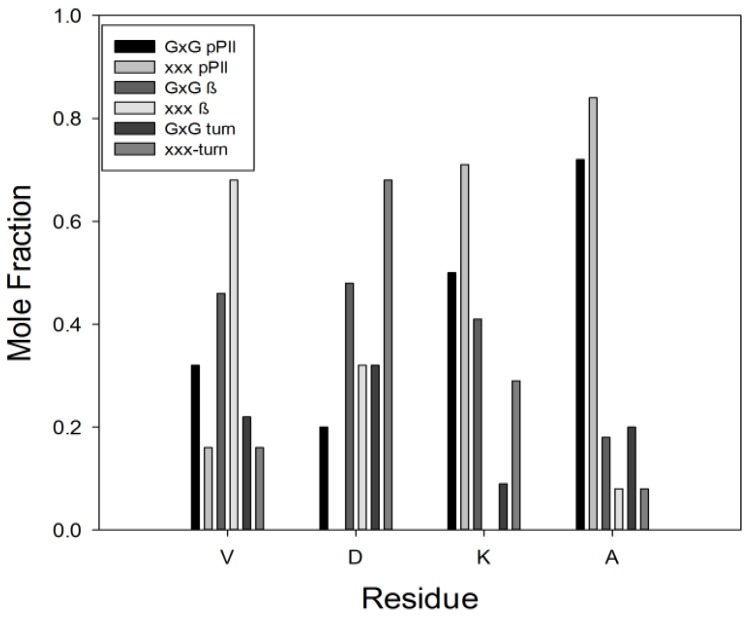
Representation of the molar fractions of amino acid residues indicated on the abscissa in GXG and XXX peptides. The code for the bars is defined in the inset of the figure. The data were taken from [138] for GAG, GVG, GDG and GKG and from [118,140] for AAA, VVV, protonated DDD and protonated KKK.

Experimental results on nearest neighbor interactions in short peptides dissolved in water are sporadic. We indicated above that alanine neighbors in AAA slightly stabilize pPII. The spectroscopic methods used to determine conformational ensembles of AAA [101] were also used to investigate VVV (trivaline), protonated trilysine (KKK) [179] as well as protonated and ionized DDD (tri-aspartic acid) [180] and compared with the respective GxG distributions. The respective fractions of the conformations of the central residues are displayed in Figure 21. Valine, for instance, as a terminal neighbor significantly increase the β-strand content of valine from 46% to 68%. For trilysine, the two neighbors stabilize what Verbaro *et al.* called a distorted pPII conformation with φ = −90° and ψ = 170° [140], whereas K adopts a much more balanced pPII/β strand in a glycine context [179]. Duitch *et al.* found that ionized DDD contains practically no pPII, *ca.* 30% β, 30% right handed helical like conformations and *ca.* 40% turn like conformations [180]. Upon side chain protonation, this all becomes straighten out and the distribution solely shows equal fractions of pPII and β-strand without any detectable turn-like conformation. In GDG, however, the protonated state shows more pPII (though β is still dominant) and less helical/turn like conformations. In the ionized state, pPII is slightly dominant (59%), coexisting with 41% of β-strand. Hence the authors concluded that ionized D as neighbor stabilizes β-strand. An earlier comparison of AFA and GFG by Pizzanelli *et al.* [181] revealed that alanine neighbors increase the pPII content of F. All these results clearly suggest in part very strong nearest neighbor effects, which are not limited to side chains with branched residues. What these data do not reveal is the different influence of N- and C-terminal neighbors. A project investigating this in detail is currently underway in our laboratory.

A more recent study conducted by Oh *et al.* combined NMR/CD analysis of a huge number of blocked tripeptides in water [141,182]. An analysis of the data was performed with a two state model in the mold of Shi *et al.* [51] thus considering solely pPII and β-strand. The spread of the obtained chemical shift and coupling data derived from NMR was reportedly narrow, which the authors attribute to small variances of the conformation distribution due to neighbors. The results led the Oh *et al.* to conclude that nearest neighbor interactions are negligible in such short peptides. This is clearly at variance with the above experimental data on tripeptides and likewise with a large body of theoretical studies. A closer inspection of the data presented by Oh *et al.* do rather suggest that nearest neighbor interactions should not be neglected, too different are conformational distributions for different neighbors. In a more recent paper from the same research group nearest neighbor influence on J-coupling constants and chemical shift is noted but not clearly assigned to any physical cause [183].

Taken together the results presented in this section provide compelling evidence for the notion that significant nearest neighbor interactions exist in unfolded peptides and proteins. However, a very clear picture has yet to emerge. Conformational and solvation effects need to be disentangled further. The same holds for the influence of N- and C-terminal neighbors. Discrepancies between coil-library distributions and residue conformations in solution are apparent and need to be investigated with regard to nearest neighbor interactions.

## 5. Conclusions and Outlook

The experimental, bioinformatic and computational results discussed in this review clearly suggest that one of the basic assumption of the classical random coil view of the unfolded state of proteins and peptides, *i.e.*, the rather unrestricted sampling of the sterically permissible sub-space of the Ramachandran plot, is an oversimplification, which does not give justice to the capability of peptide/protein—water interactions to restrict and diversify the conformational propensities of amino acid residues. With the exception of glycine residues, the distribution of which has only been sporadically investigated, all residues have in common that they exhibit a rather strong preference for extended pPII and/or β-strand conformations. The remaining fraction is generally distributed over several turn-like conformations with right-handed helical conformations just as one of the possible options. Amino acid residues with short side chains and hydrogen bonding capacity exhibit an above the average population of such more compact structures, [77]. The pPII/β manifold of residues differ in terms of the population ratio of pPII and β-strand conformations as well as with regard to the positions of the respective sub-populations. Generally the pII/β-distributions seem to be narrower than they would be if solely determined by steric interactions. Alanine has been experimentally established as exceptional owing to its way above average pPII propensity (0.72 in GAG, 0.84 in AAA) [51,120,138]. Residues with Cβ-branched side chains exhibit a slight preference for β-strand. Most of the remaining amino acid residues (aspartic acid being an exception) exhibit some preference for pPII over β, with the pPII-β equilibrium dominating the conformational ensemble as a whole. Data from coil libraries, computational studies, and a limited set of data on short peptides suggest a strong influence of nearest neighbors on amino acid residue propensities, at variance with another pillar of the random coil theory, the so called isolated pair hypothesis [81,82,83,85,101,179,180,183,184]. A more detailed assessment of these interactions for unfolded peptides in solution is an ongoing process in our laboratory.

A very recent thermodynamic study by Toal *et al.* [167], which has not yet been discussed, indicates that the conformational equilibrium between pPII and β-strand is subject to a nearly ideal enthalpy-entropy compensation. This means that rather large enthalpic (favoring pPII) and entropic contributions (favoring β-strand) far exceed the respective Gibbs energy and that they differ substantially between different amino acid residues. The authors attributed this compensation mainly to modification of peptide hydration by side chains, but to a minor extent (for V and I) also different sampling of rotamers in pPII and β. In addition, the authors identified iso-equilibria for two subsets of residues at 297 and 305 K. This data suggests that although different amino acid residues indeed have variant conformational preferences in the unfolded state, within the found temperature regime ensembles may differ solely with respect to their capability to adopt turn-like conformations. It needs to be seen how this picture is modified in the presence of nearest-neighbor interactions.

Now the question arises whether or not all these results are really relevant for the unfolded states of longer peptides and proteins. One assumption underlying the random coil model is that water acts as a good solvent thus maximizing the number of peptide/protein-water contacts. If this were indeed the case individual propensities obtained from short peptide studies would be directly applicable to unfolded states of, e.g., proteins. However, as already mentioned in the introduction, ample experimental evidence challenges also this aspect of the random coil model. Particularly NMR studies of Blackledge, Schwalbe and their respective colleagues revealed local order in IDPs and unfolded states of foldable proteins alike, which reflect the presence of non-local, particularly hydrophobic interactions [16,17,18,19,44,45]. Local order can involve the temporary formation of helices and even β-strands. A similar view has emerged from computational studies of different amyloid β fragments. IDPs themselves show a rather large disparity of disorder. This led Uversky to classify them in categories called molten globule, pre-molten globule and coil [1]. With respect to the protein folding problem these insights reveal a second source of entropy reduction in addition to the conformational propensities on which this review focuses.

What could now be the relevance of conformational propensities for the structure analysis of unfolded proteins/peptides and IDPs? First, all the thus far available data on conformational propensities can facilitate the assessment of the unfolded states’ maximal conformational entropy. Second, they should be used to define a reference system to which results for the structure analysis of larger system should be compared. With regard to NMR this is currently done by using mostly chemical shifts and to a lesser extent coupling constants of short glycine based peptides as so called statistical coil reference. This strategy might have its shortcomings. Avbelj recently showed that chemical shifts of, e.g., amide protons in short peptides are determined by the respective solvent accessibility, which itself is side chain dependent [74]. If, as experimental data suggest, amino acid residues can get desolvated, a change of their chemical shift will be induced. Hence, observed shift changes of amide protons in larger peptides and proteins would reflect both, reduced solvation and structural changes. With regard to coupling constants our own experience teaches us that relying just on one or two constants might not lead to reliable results. We therefore propose an alternative, namely a structural analysis of IDPs and proteins based on a variety of spectroscopic data and a direct comparison with predictions based on the structural analysis of short peptides. This strategy could include vibrational spectroscopy, for which we recently showed that different mixtures of locally ordered oligopeptides can indeed be distinguished mostly by means of the VCD signal of the amide I mode [185]. However, there is good reason to believe that the solvent sensitivity of, e.g., side chain Raman bands should be utilized as well [186].

While the above NMR studies focus on local order caused by the partial desolvation of amino acid side chains in proteins, a recent study by Schwalbe *et al.* on two IDPs, the monomers of hTau 40 and α-synuclein, surprisingly revealed segments of these proteins that show a rather surprising large sampling of pPII like conformations [187]. In line with earlier hypotheses put forward by Barron and coworkers [188,189,190] based on ROA studies on several self-aggregating unfolded proteins they found that these segments to be pivotal for self-aggregation into soluble oligomers. The authors went so far to propose some type of cooperativity that stabilizes this conformational preference. Thus far, even those groups whose work provided strong evidence for the preponderance of pPII in unfolded peptides have found no evidence for any type of cooperativity (nearest neighbor interactions does not automatically lead to cooperativity) [184]. However, H NMR studies on Aβ-fragments by Danielsson *et al.* indicate indeed some pPII-related cooperativity [191]. These authors’ paper as well as vibrational spectroscopy studies of monomeric Aβ_1–28_ also suggest a disproportional fraction of pPII [192]. All these results seem to indicate that pPII propensities of amino acid residues in some IDPs might be larger than their intrinsic propensity and that their underlying determinants therefore deserve some attention even in the context of the structural analysis of large unfolded proteins.

At the beginning of this article we differentiated between local and global random coil behavior. We defined local random coil on the sub-nano scale residue level as the neighbor independent conformational sampling of residues solely restricted by steric accessibility. Global random coil behavior on a nanoscale, however, is describable as being consistent with prediction from polymer theory with regard to the number of residue dependence of the, e.g., the radius of gyration. Does the breakdown of the local random coil model automatically imply that the global random coil model shouldn’t work? The answer is a clear no. At a very early stage of the pPII debate, when, e.g., the results of Shi *et al.* were sometimes interpreted as being indicative of pPII helix like structures in unfolded peptides and proteins, Fitzkee and Rose showed convincingly that protein ensembles containing largely native protein structures connected with disordered links solely governed by hard sphere sterics show global random coil behavior with regard to end-to-end distances and radii of gyration [193]. Since the conformational preferences discussed in this article do by no means indicate the existence of such ordered motifs like stable pPII helices in the unfolded state, their existence should by no means interpreted as being inconsistent with the global random coil model. As a matter of fact, the result of Fitzkee and Rose strongly imply the necessity for the differentiation between local and global random coil, as done in this review.

This article has provided several lines of evidence for the necessity of avoiding the term random coil for unfolded peptides and proteins, at least with regard to the local level. Small angle X-ray scattering results of Kohn *et al.* suggest that it is not even always applicable on a global level [194]. What would be an alternative terminology? At a very early stage Scheraga and colleagues used the term statistical coil, which reflects the fact that the Gibbs energy landscape of residues in unfolded proteins might vary on the order of RT [134,135]. Though we think that this is an improvement over the random coil terminology, the definition is still somewhat fuzzy since it does not set an exact upper limit. Can we still use the term statistical coil if the difference between two conformations exceeds 2RT? That’s the situation for alanine. Moreover, the Gibbs energy difference between turn-like and extended structures is mostly larger than 2RT, which restricts the usage of “statistical coil” to the pPII/β-region of a majority though not all peptides. We think that the recently emerging term disordered is more appropriate in that it does not make any assumptions about distributions but reflects the fact that even in the “new view” residues still fluctuate rather quickly between different conformations. What is at stake now for future research is to develop quantitative measures of disorder which avoids an “all cats are gray in the dark” like thinking about proteins.
